# Tannic acid-iron stabilized probiotic silver nano hybrids: Multi-target gut microbiota modulation and intestinal barrier restoration

**DOI:** 10.1016/j.mtbio.2025.102106

**Published:** 2025-07-16

**Authors:** Saisai Gong, Zhibo Zeng, Mingjue Liu, Xianfu Wang, Chuxian Quan, Muhammed Farhan Rahim, Yaping Wang, Aoyun Li, Md. F. Kulyar, Zhexue Lu, Jiakui Li

**Affiliations:** a College of Veterinary Medicine, Huazhong Agricultural University, Wuhan, China; b College of Chemistry, Huazhong Agricultural University, Wuhan, China; c College of Animals Husbandry and Veterinary Medicine, Tibet Agriculture and Animal Husbandry University, Linzhi, China; d College of Veterinary Medicine, Henan Agricultural University, Zhengzhou, China; e College of Animal Science and Technology, Henan Agricultural University, Zhengzhou, China

**Keywords:** Probiotics, Silver nanoparticles, Gut microbiota, Intestinal inflammation, Nanomedicine, Bio-nanocomposites

## Abstract

The limited efficacy and adverse effect profile of current pharmacological treatments for intestinal inflammation underscore the need for modalities that preserve gut microbiota balance while attenuating inflammation. The aim of this study was to develop and evaluate a BL@TA-Fe^III^@AgNPs system with a view to provide synergistic efficacy against intestinal injury. This research introduces an innovative hybrid bio nanocomposite, BL@TA-Fe^III^@AgNPs, comprising viable *Bacillus licheniformis* coated with a tannic-acid/Fe^III^ coordination layer that nucleates and anchors 7 ± 1.5 nm silver nanoparticles. Characterization of this composite material was performed using TEM, EDS, XRD, and XPS. Functional assays included probiotic viability, tolerance to simulated gastric and intestinal fluids, and bactericidal activity against *Escherichia coli* and *Staphylococcus aureus*. In-depth safety evaluations were carried out using both cell cultures and a mouse model. Therapeutic effects in an acute LPS-endotoxemia mouse model were analyzed by 16S rRNA gene amplicon sequencing and untargeted LC-MS/MS metabolomics of cecal contents. Characterization confirmed structural integrity, colloidal stability in physiological media, and low cytotoxicity (IC_50_ > 100 μg Ag mL^−1^). BL@TA-Fe^III^@AgNPs restored transepithelial electrical resistance, lowered malondialdehyde levels, and reshaped microbiota composition and metabolite networks relative to LPS controls. Restoration of the Firmicutes: Bacteroidetes ratio and elevated short chain fatty acid concentrations support BL@TA-Fe^III^@AgNPs as a promising adjunctive strategy for acute endotoxin-induced intestinal injury.

## Graphical abstract

**Graphical abstract** Protective effect of BL@TA-Fe^III^@AgNPs against LPS-induced damage. The BL@TA-Fe^III^@AgNPs complex orchestrates the remediation of lipopolysaccharide (LPS)-induced damage via a multifaceted approach: it enhances hepatic oxidative resilience and reinstates antioxidant capacity, restores the structural integrity of the intestinal barrier, alleviates dysbiosis of the gut microbiota, and optimizes intestinal metabolism, all while suppressing the expression of inflammatory cytokines.

**Figure:**
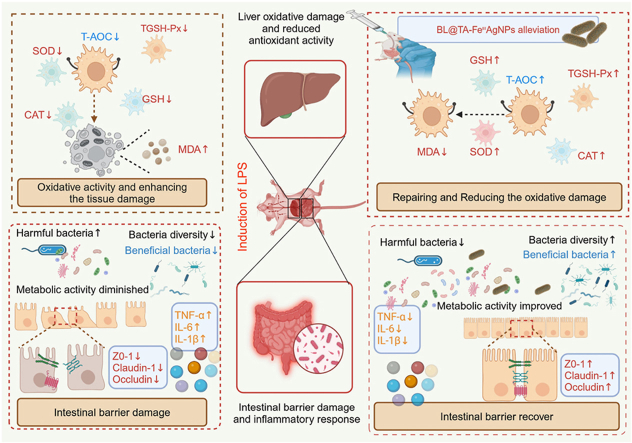

## 1 Introduction

Intestinal inflammation, a hallmark of conditions such as enteritis and ulcerative colitis, manifests through symptoms such as diarrhea and abdominal discomfort. In addition to clinical symptoms, inflammation disrupts the gut microbiota, exacerbating disease progression and impairing overall health [1]. Current treatments, predominantly antibiotics, anti-inflammatory agents, and immunosuppressants, face limitations such as drug resistance and adverse impacts on the gut microecology [[2], [3], [4], [5], [6]]. To address these issues, nanoparticle-based strategies have emerged as promising alternatives, suggesting potential in modulating the gut microbiota and mitigating resistance mechanisms [7,8]. Despite their promise, challenges persist, including improving site-specific accumulation, minimizing self-aggregation, and reducing off-target effects.

Recent advances in biotechnology highlight bacteria's natural tropism for inflammatory or tumorous sites, presenting an innovative avenue for targeted therapy [[9], [10], [11]]. For example, Shewanella oneidensis MR-1, integrated with doxorubicin-loaded MIL-101 nanoparticles, has been shown to enhance chemotherapeutic efficacy by exploiting bacterial colonization capabilities [9]. In parallel with these developments, probiotics have garnered significant attention for their ability to maintain gut homeostasis [12,13]. Engineered probiotics tailored for intestinal colonization [[14], [15], [16]], have been shown to resist gastrointestinal adversities and provide multifaceted benefits, such as pathogen inhibition, oxidative damage reduction, and immune modulation including macrophage polarization to restore barrier integrity [[17], [18], [19]]. Our group has identified several robust probiotics, including *Bacillus amyloliquefaciens*, *Bacillus licheniformis*, *Bacillus subtilis*, and *Lactobacillus reuteri*, which suggest potent anti-inflammatory and immunomodulatory properties, positioning them as viable carriers for advanced therapeutic delivery systems [[20], [21], [22], [23]]. On the basis of these findings, developing novel enteric probiotics as biofunctional carriers that target the gut is a promising application strategy.

Silver nanoparticles (AgNPs), renowned for their potent antimicrobial properties, offer a complementary strategy for addressing intestinal inflammation [[24], [25], [26]]. AgNPs have shown a potential to mitigate tissue damage [[27], [28], [29]], by disrupting microbial membranes and attenuating inflammatory responses. Additionally, AgNPs suppress the inflammatory response and minimize the degree of tissue damage associated with intestinal inflammation [30,31]. However, their clinical utility is constrained by their non-specificity and aggregation in vivo. To overcome these challenges, we propose a novel Probiotic@TA-Fe^III^@AgNPs system, which that synergizes the gut-targeting attributes of probiotics with the stabilizing properties of tannic acid for AgNPs. This design ensures targeted delivery to inflamed gastrointestinal sites, overcoming the conventional limitations of nanoparticle therapy. Also, our findings are restricted to LPS-driven acute injury; efficacy under chronic DSS or TNBS colitis and adaptive immune engagement has yet to be tested. Future investigations will evaluate the therapeutic efficacy and microbiota-modulating potential of this system in LPS-induced inflammation models. Integrating nanotechnology with probiotics represents a transformative approach to treating intestinal inflammation, paving the way for innovative therapeutics that address current limitations while advancing the interplay between the gut microbiota and nano-based therapies.

## 2 Materials and methods

### 2.1 Materials

The reagents employed in this study include tannic acid, ferric chloride (FeCl_3_·6H_2_O), and MOPS buffer, which were procured from Shanghai Aladdin Biochemical Technology Co., Ltd. Potassium carbonate (K_2_CO_3_) was sourced from Sinopharm Chemical Reagent Co., Ltd. Silver nitrate (AgNO_3_) was provided by Shanghai Lingfeng Chemical Reagent Co., Ltd. A Biosharp cell counting kit (CCK-8) was obtained from Beijing Labgic Technology Co., Ltd. A commercial ELISA kit for murine interleukin-1β (IL-1β) was acquired from Wuhan Zokeyo Biotechnology Co., Ltd. Reagents for superoxide dismutase (SOD), malondialdehyde (MDA), glutathione (GSH), total antioxidant capacity (T-AOC), catalase (CAT), and glutathione peroxidase (GSH-Px) were purchased from Abbkine Scientific Co., Ltd., Wuhan, China. Bacterial culture media were obtained from Qingdao Hi-Tech Industrial Park Hope Bio-Technology Co., Ltd. The biochemical detection reagents were supplied by Shenzhen Mindray Bio-Medical Electronics Co., Ltd. All primer sequences were designed and synthesized by Shanghai Sangon Biotech Co., Ltd. The biological antibodies were procured from Wuhan Proteintech Group, Inc. All other reagents used in this study were of analytical grade and purchased from commercial suppliers.

### 2.2 Bacterial strains and culturing

*Bacillus licheniformis* (BL) *and Lactobacillus reuteri* (LR) strains were isolated and identified by our research team from the intestinal environment of yaks in Tibet, China. A colony from the preserved strains was selected and cultured in 50 mL of LB or MRS medium at 37 °C with constant shaking at 150 rpm for 10 h to reach the stationary growth phase.

### 2.3 Growth curve determination and colony forming unit (CFU) counting of BL strains

A single colony of the BL strain was aseptically selected from the preserved agar plate and inoculated into 50 mL of sterile LB broth medium. The culture was incubated at 37 °C with continuous shaking at 150 rpm. To establish the growth kinetics, the optical density at 600 nm (OD_600_) was measured at 2-h intervals over a 24-h period using a spectrophotometer. For colony enumeration, 100 μL of bacterial suspension harvested during the stationary growth phase was serially diluted 10-fold in sterile phosphate-buffered saline (PBS). Aliquots (100 μL) of each dilution were spread uniformly onto fresh LB agar plates and incubated at 37 °C for 18–24 h. The colonies were counted manually and the concentration of viable bacteria was calculated.

### 2.4 Synthesis and characterization of BL@TA-Fe^III^@AgNPs

A total of 15 mL of cultured BL bacterial suspension was centrifuged to remove the supernatant, and the bacterial pellet was washed three times. The resulting pellet was then diluted to 4 mL with H_2_O, mixed with 1 mL of tannic acid (TA) solution at a concentration of 2 mg/mL, and thoroughly agitated for 30 s. Next, 1 mL of ferric chloride hexahydrate (FeCl_3_·6H_2_O) solution at a concentration of 0.5 mg/mL was added, and the mixture was shaken again for 30 s. Next, 2 mL of MOPS buffer (20 mmol/L, pH 7.4) was rapidly added, and the mixture was stirred for 2 min. The TA-Fe^3+^-coated bacteria (BL@TA-Fe^Ⅲ^) were then uniformly dispersed in 2.5 mL of water and adjusted to a pH of approximately 7.5 with 2.5 mmol/L potassium carbonate (K_2_CO_3_). Finally, 500 μL of silver nitrate (AgNO_3_) solution at a concentration of 5 mmol/L was added, and the reaction was allowed to proceed for 1 h to prepare BL@TA-Fe^Ⅲ^@AgNPs. The morphological characteristics of BL@TA-Fe^Ⅲ^@AgNPs and the attachment of the silver nanoparticles were observed via transmission electron microscopy (TEM). Energy-dispersive X-ray spectroscopy (EDS) was employed to analyze the elemental composition of the composite material. X-ray diffraction (XRD) was used to assess the crystallinity of the silver nanoparticles. In contrast, X-ray photoelectron spectroscopy (XPS) provided insights into the chemical composition and electronic state of the composite material. Finally, the charge characteristics of the composite material were assessed via a zeta potential analyzer to evaluate its stability in physiological environments.

### 2.5 Analysis of BL activity in BL@TA-Fe^III^@AgNPs

1 mL of the BL, BL@TA-Fe^Ⅲ^, or BL@TA-Fe^Ⅲ^@AgNPs suspension was added to 50 mL of liquid culture medium and incubated at 37 °C with shaking at 200 rpm. Every 2 h, the optical density at 600 nm (OD_600_) of the bacterial suspension was recorded, resulting in the generation of a bacterial growth characteristic curve over a 24 h. This curve was used to analyze the dynamic features during the lag and stationary phases. To accurately quantify the number of surviving bacteria during the stationary phase: during the stationary phase (as determined by the OD_600_ plateau), 100 μL of the culture was serially diluted 10-fold in six dilution gradients (10^−1^ to 10^−6^) with sterile PBS(pH 7.4). Triplicate aliquots (100 μL) of the 10^−4^, 10^−5^, and 10^−6^ dilutions were spread onto LB agar plates. The plates were incubated at 37 °C for 18 h and colonies were counted manually.

### 2.6 Resistance of BL@TA-Fe^III^@AgNPs to the gastrointestinal environment

After centrifugation, 1 mL of the BL@TA-Fe^Ⅲ^@AgNPs suspension was washed three times with PBS and subsequently treated with simulated gastric fluid (SGF) for 2 h or simulated intestinal fluid (SIF) for 4 h, after which the mixtures were incubated at 37 °C with shaking at 200 rpm. Viable bacterial counts were determined via a dilution-plating method. Additionally, TEM and EDS analyses were conducted to observe the morphological changes in the composite material and the integrity of its layered structure following treatment with the simulated gastrointestinal fluids.

### 2.7 Analysis of the bactericidal effect of BL@TA-Fe^III^@AgNPs

1 mL of the BL@TA-Fe^Ⅲ^@AgNPs suspension was mixed with 1 mL of both the *Escherichia coli* and *Staphylococcus aureus* suspensions, and the mixture was placed in an anaerobic incubator at 37 °C with shaking at 200 rpm for 4 h. Following the incubation, 100 μL of the mixed solution was spread onto tryptone bile salt X-glucuronide (TBX) agar plates (which selectively indicate E. coli with blue-green colonies) and onto specific growth media for *Staphylococcus aureus* (which produces reddish-purple colonies). After 24 h of incubation, the colonies were observed and counted to evaluate the bactericidal efficacy of the BL@TA-Fe^Ⅲ^@AgNPs.

### 2.8 In vitro antioxidant activity and multi-enzyme activity assessment

ABTS radical scavenging assay: ABTS radical scavenging capacity was determined via a total antioxidant capacity assay kit (Macklin, Cat# T931096) following the manufacturer's instructions. The samples included a blank control (negative), BL, BL@TA-Fe^III^, and BL@TA-Fe^III^@AgNPs at concentrations of 1 × 10^4^, 1 × 10^5^, 1 × 10^6^, 1 × 10^7^, and 1 × 10^8^ particles/mL, respectively. After the reaction was complete, the mixtures were centrifuged, and the supernatant absorbance was measured at 734 nm.

DPPH radical scavenging assay: DPPH (2,2-diphenyl-1-picrylhydrazyl; Macklin, Cat# D807297) was used as the radical source. The samples (negative control, BL, BL@TA-Fe^III^, and BL@TA-Fe^III^@AgNPs at 1 × 10^4^–1 × 10^8^ particles/mL) were assessed as follows: 2 mL of sample was added to 1 mL of 0.2 mmol/L DPPH solution. For the negative control (A_negative), 2 mL of ultrapure water was used instead of the sample. The blank control (A_blank) contained 2 mL of sample and 1 mL of absolute ethanol. The reaction mixtures were incubated in the dark at 37 °C for 30 min with shaking, and then centrifuged, and the supernatant absorbance was measured at 517 nm. DPPH scavenging activity (%) was calculated as follows: scavenging (%) = [(A_negative − (A_sample − A_blank))/A_negative] × 100.

SOD-like activity (O_2_- scavenging): Superoxide anion (O_2_-) scavenging activity, indicative of SOD-like behavior, was evaluated via electron spin resonance (ESR) spectroscopy (Bruker A300-10/12, Germany) via the xanthine/xanthine oxidase system. The control mixture contained 20 μL of 10 mM xanthine (in PBS), 20 μL of 1 U/mL xanthine oxidase (in PBS), 10 μL of 200 mM BMPO spin trap (Sigma), and 50 μL of PBS buffer and was incubated for 10 min before ESR measurement. For the test samples, 50 μL of sample mixture was replaced with 50 μL of PBS buffer.

CAT-like activity (H_2_O_2_ scavenging): Catalase (CAT)-like activity was assessed via H_2_O_2_ decomposition monitored by ESR using CTPO (3-carbamoyl-2,2,5,5-tetramethyl-3-pyrroline-1-yloxyl, Sigma) as the spin probe. The control mixture comprised 500 μL of 100 mM CTPO, 400 μL of 20 mM H_2_O_2_, and 100 μL of deionized water and was deoxygenated for 30 min. In the test mixture, 100 μL of sample mixture was replaced with deionized water.

POD-like activity (TMB oxidation): The peroxidase (POD)-like activity of BL@TA-Fe^III^ and BL@TA-Fe^III^@AgNP (1 × 10^6^, 1 × 10^7^, and 1 × 10^8^ particles/mL) was analyzed via 3,3′,5,5′-tetramethylbenzidine (TMB) as a chromogenic substrate in 0.1 M sodium acetate buffer (pH 4.5) or simulated intestinal fluid (pH 7.4) containing 0.5 mM H_2_O_2_. The reaction mixture (700 μL of buffer) contained 50 μL of 10 mM H_2_O_2_ and 200 μL of 0.01 % (w/v) TMB solution. The nanoparticles were added last to initiate the reaction. After gentle vortex mixing, the reactions proceeded at 37 °C in the dark. Aliquots were withdrawn at t = 0, 1, 3, 5, 10, and 15 min and quenched immediately with 250 μL of 2 M H_2_SO_4_ (final volume of 1.25 mL). The quenched solutions were centrifuged, and 1 mL of the supernatant was transferred to a cuvette to measure the absorbance at 450 nm (OD_450_).

### *2.9* *In vitro* safety evaluation of BL@TA-Fe^III^@AgNPs

IPEC-J2 cells were seeded into a 96-well plate at a density of 5 × 10^3^ cells per well. Pre-synthesized BL@TA-Fe^III^@AgNPs (equivalent to 19.70 ± 0.27 μg Ag mL^−1^, diameter 7.11 ± 1.54 nm) were dispersed in complete DMEM and applied to IPEC-J2 cells for 12 h. Cell viability was then measured by adding 10 μL CCK-8 reagent once per well and reading absorbance at 450 nm after a 2 h incubation, following the manufacturer's single endpoint protocol.

### 2.10 *In vivo* safety evaluation of BL@TA-Fe^III^@AgNPs

To further investigate the biosafety of BL@TA-Fe^Ⅲ^@AgNPs, we designed a 21-day intervention experiment using a mouse model. A total of 40 mice (20 males and 20 females, aged 6–8 weeks) were randomly divided into four groups: a control group (C) and three dosage groups based on viable BL counts, low (1 × 10^7^ CFU + 0.8 μg Ag), medium (1 × 10^8^ CFU + 8 μg Ag), and high (1 × 10^9^ CFU + 80 μg Ag), with silver doses verified by ICP-MS. The dose of BL was determined based on a previous study [32]. We monitored the general appearance and body weight changes of the mice in each group. The mice were euthanized at the end of the 21 days, and whole blood and serum samples were collected. Additionally, tissue samples from the liver, kidneys, spleen, testes, and intestines were harvested and fixed in a tissue fixation solution for further analysis.

### 2.11 Evaluation of the metabolism and absorption safety of BL@TA-Fe^III^@AgNPs

To evaluate the metabolic transfer of the metal elements silver (Ag) and iron (Fe) within BL@TA-Fe^Ⅲ^@AgNPs, an additional group of mice (18 total, with equal numbers of males and females, aged 6–8 weeks) was established and administered the treatment according to medium dosage conditions (1 × 10^8^ CFU of BL@TA-Fe^Ⅲ^@AgNPs). For intestinal retention analysis, intestinal contents were collected seven days after gavage and analyzed by ICP-MS to quantify residual Fe and Ag. Serum, fecal, and liver tissue samples were collected at intervals of seven days during the intervention period. Following the cessation of treatment, samples were collected for an additional two weeks. The samples were then analyzed via ICP-MS to confirm the metabolic status of the metal components within the body.

### 2.12 Animal experimental design and sample collection

A total of 60 male Kunming mice weighing 20 ± 2 g were purchased from the Experimental Animal Center of Huazhong Agricultural University and divided equally into five groups: the control group (C), model group (M), and treatment group (BL, TA@AgNPs, and BL@TA-Fe^III^@AgNPs). After one week of adaptation, mice in the M, BL, TA@AgNPs, and BL@TA-Fe^III^@AgNPs groups were injected intraperitoneally with 5 mg/kg LPS (Beyotime Biotechnology, ST1470) for five consecutive days [33], whereas the mice in the C group were injected with saline. *Bacillus licheniformis suspension* (10^8^ CFU, 0.4 mL) was administered to the mice in the BL group starting on day six, whereas animals in the BL@TA-Fe^III^@AgNPs groups received equivalent volumes of BL@TA-Fe^III^@AgNPs (10^8^ CFU, Ag 19.70 ± 0.27 μg/ml, 0.4 mL) suspensions. In addition, the TA-Fe^III^@AgNPs group was administered a TA-Fe^III^@AgNPs suspension (Ag 13.13 ± 0.71 μg/ml, 0.6 mL) with the same silver content as a separate Ag intervention control. The silver content in the suspensions was confirmed via ICP-MS analysis to ensure the accuracy of the administered doses. The mice in the C and M groups were gavaged with distilled water. The intervention lasted 20 days, during which the body weight was observed and recorded. On the last day of the experiment, the mice were sacrificed, the serum was collected and stored at −20 °C, and the small intestinaland cecum contents were collected. After rapid freezing in liquid nitrogen, the samples were stored at −80 °C.

All animals involved in this study were allowed free access to feed and water in a comfortable environment. The research protocol was approved by the Institutional Animal Care and Ethical Committee of Huazhong Agricultural University (Ethical Numbers: HZAUMO-2024-0367 and HZAUMO-2023-0277) and was conducted according to the guidelines set forth by the National Institutes of Health's Guide for the Care and Use of Laboratory Animals, the Animal Welfare Act, and the regulations established by the Ministry of Science and Technology of the People's Republic of China.

### 2.13 Hematological and serum analysis

Whole blood samples were analyzed for their cellular composition via a hematology analyzer (Mindray BC-5000 Automated Hematology Analyzer). Serum samples were processed according to the manufacturer's instructions to determine the levels or activities of superoxide dismutase (SOD), malondialdehyde (MDA), glutathione (GSH), total antioxidant capacity (T-AOC), interleukin-1 beta (IL-1β), and immunoglobulin G (IgG). Additional serum biochemical parameters, including total protein (TP), albumin (ALB), alkaline phosphatase (ALP), alanine aminotransferase (ALT), aspartate aminotransferase (AST), urea (UREA), creatinine (CREA), uric acid (UA), glucose (Glu), total cholesterol (TC), triglycerides (TG), and electrolyte levels such as calcium (Ca), phosphorus (P), and magnesium (Mg), were measured via an automatic biochemical analyzer (Mindray BS-240 VET Automated Biochemical Analyzer).

### 2.14 Histopathological examination

Tissue samples were fixed in a 4 % paraformaldehyde solution for 24 h, followed by embedding in paraffin to prepare sections. These sections were subsequently stained with hematoxylin and eosin (H&E) to observe and examine the morphological characteristics of the tissues. In addition, bowel injury was assessed using a modified version of the Erben Bowel Injury Scoring System. The detailed scoring criteria are shown in Table S1.

### 2.15 qPCR analysis

To quantify the expression levels of target genes, dye-based quantitative PCR (qPCR) was employed. Total RNA was extracted from the samples via TRIzol reagent according to the manufacturer's instructions. The purity and concentration of RNA were assessed via a Nano-1000 micro-spectrophotometer (Hangzhou Youmi Instruments Co., Ltd.). For cDNA synthesis and subsequent qPCR, we utilized the HiScript II Q RT SuperMix for qPCR (+gDNA wiper) kit and the Taq Pro Universal SYBR qPCR Master Mix (Nanjing Vazyme Biotech Co., Ltd.), following the manufacturer's guidelines. The housekeeping gene β-actin was used as an internal control to normalize the gene expression data. Fold changes in gene expression were calculated via the 2^-ΔΔC method, and all reactions were performed in triplicate to obtain mean values. Statistical analyses were conducted via Student's t-test, with a significance threshold set at p < 0.05. The primer sequences utilized in the experiment are listed in Table S2.

### 2.16 Western blot analysis

After the intestinal tissue samples were retrieved from the −80 °C freezer, they were promptly homogenized in RIPA buffer containing protease and phosphatase inhibitors via a tissue grinder. The lysates were subsequently centrifuged at 10,000×*g* for 15 min at 4 °C to remove cellular debris. The protein concentration of the supernatants was measured via the enhanced BCA assay. Equal amounts of protein samples were separated by SDS-PAGE and transferred to a PVDF membrane via a Bio-Rad transfer apparatus. After the membrane was blocked with 5 % non-fat milk in TBST buffer, it was incubated overnight at 4 °C with the primary antibody, followed by a 1-h incubation at room temperature with the HRP-conjugated secondary antibody. Detection was performed using an ECL substrate, and imaging was conducted with a Bio-Rad ChemiDoc XRS + system. The intensities of the protein bands were quantified via ImageJ software, and the protein expression levels were normalized to those of β-actin. The antibody information used in this experiment is listed in Table S3.

### 2.17 Gut microbiota analysis

Bacterial DNA was extracted from the fecal pellets via the DNeasy PowerSoil kit (Qiagen, Germany) according to the manufacturer's instructions. The V3-V4 variable region of the bacterial 16S rRNA gene was amplified via PCR using the primers (343F: 5′-TACGGRAGGCAGCAG-3’; 798R: 5′-AGGGTATCTAATCCT-3′). The PCR products were purified with the QIAquick gel extraction kit and sequenced on the Illumina MiSeq platform (Illumina Inc., USA). The sequences were clustered into operational taxonomic units (OTUs) at a 97 % similarity threshold, and various indices were used to analyze their alpha and beta diversity. Significant differences in microbial community abundance were evaluated using t-tests, while Spearman's rank correlation analysis was conducted to explore interspecies interactions. Data processing was facilitated by BMKCloud (www.biocloud.net). To predict the functional gene repertoire of each sample we ran PICRUSt2 v2.5.1 with default NSTI filtering and Genome Taxonomy Database release R202. Kyoto Encyclopedia of Genes and Genomes Orthologue tables were collapsed to MetaCyc pathway level and differentially abundant pathways were identified with ALDEx2 [34,35].

### 2.18 Gut metabolomics analysis

The 50 mg of cecal content were mixed with 1000 μL of extraction solution, vortexed for 30 s, and subjected to ball milling at 45 Hz for 10 min, followed by ultrasonic treatment in an ice-water bath for another 10 min. Metabolites were detected using a liquid chromatography-tandem mass spectrometry (LC-MS/MS) system, consisting of a Waters Acquity I-Class PLUS ultra-high-performance liquid chromatograph and a Waters Xevo G2-XS QTOF mass spectrometer, which employs a Waters Acquity UPLC HSS T3 column (1.8 μm, 2.1 × 100 mm). Mass spectrometry data were collected via MassLynx V4.2, processed with Progenesis QI, and metabolite identification involved the METLIN database and public repositories. Principal component analysis (PCA) was used to assess metabolic variations, and a volcano plot was generated to illustrate differences between groups. Significantly different metabolites were annotated and enriched via the KEGG database, and a network diagram was constructed to explore their biological functions. Data processing was conducted with BMKCloud (www.biocloud.net).

### 2.19 Correlation analysis

To examine the relationship between gut microbiota abundance, metabolite content, and the expression of intestinal damage and infection marker genes, we used the OriginPro 2021 software program for data analysis. Additionally, Pearson correlation analysis was employed to generate correlation heat maps.

### 2.20 Statistical analysis

The expressed values are represented as means ± standard deviation (SD). Group differences were assessed by one way ANOVA followed by Tukey's honestly significant difference (HSD) post hoc test. Statistical significance was set at *p* < 0.05 (two-tailed). Statistical analyses were performed in SPSS Statistics v21 (IBM Corp.); graphs were generated in GraphPad Prism v7 (GraphPad Software). Assumptions were checked with the Shapiro Wilk test and Q-Q plot inspection for normality, and Levene's test for homogeneity of variances. All datasets satisfied both criteria (*p* > 0.05); if either assumption was violated, the comparison was rerun with Welch ANOVA and Games-Howell post-hoc. Counterfactual mediation was run in PROCESS v4.1 (model 4, 5000 bias corrected bootstraps) with Intestinimonas abundance as mediator and FITC-dextran permeability as outcome.

## 3 Results and discussion

### 3.1 Synthesis and characterization of Probiotic@TA-Fe^III^@AgNPs

Previous studies conducted by our group have suggested the exceptional tolerance of *Bacillus licheniformis* (BL)to the complex gastrointestinal environment and its ability to ameliorate intestinal diseases through effective colonization [32]. Therefore, BL was selected as a representative strain for synthesizing the Probiotic@TA-Fe^III^@AgNPs composite in this study. In addition, under the established culture conditions, BL reached proliferation stabilization at 10 h, when the bacterial concentration was 3.3 × 10^8^ CFU/ml (Fig. S1). This result confirms the controllability and reproducibility of BL concentration during material preparation.

**Fig. 1:**
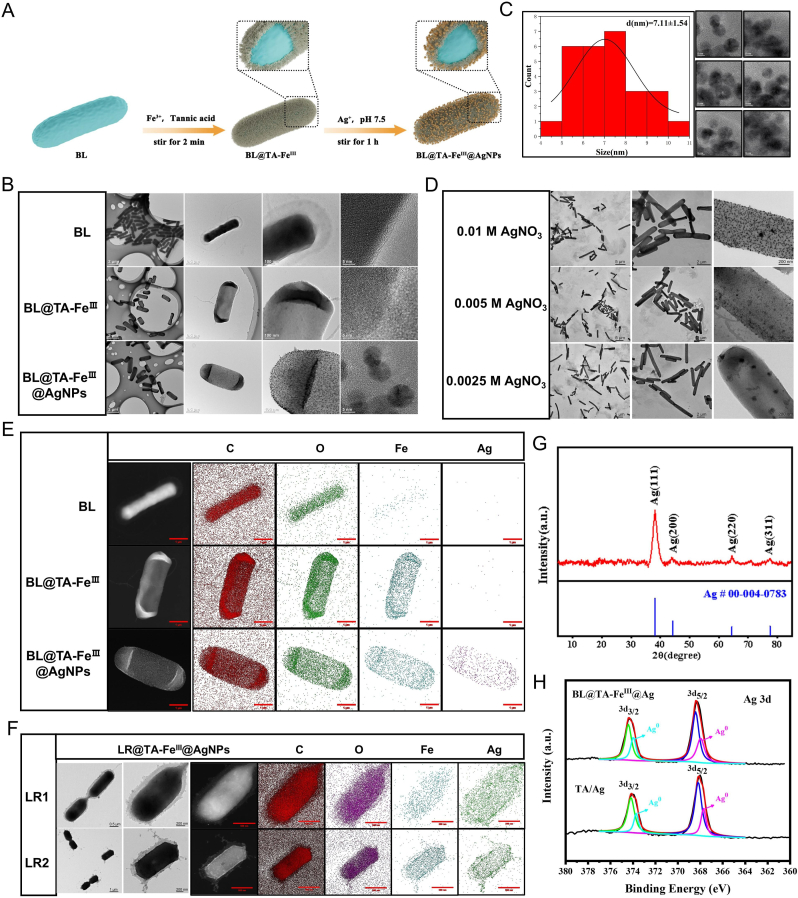
The construction and morphological characterization of BL@TA-Fe^III^@AgNPs. (A) Schematic of the synthesis of BL@TA-Fe^III^@AgNPs. (B) TEM images of BL, BL@TA-Fe^III^, BL@TA-Fe^III^@AgNPs. (C) Particle size distribution of nanoparticles on the surface of BL@TA-Fe^III^@AgNPs. (D) TEM images of BL@TA-Fe^III^@AgNPs prepared with different concentrations of AgNO_3_. (E) EDS elemental analysis patterns of BL, BL@TA-Fe^III^, BL@TA-Fe^III^@AgNPs. (F) TEM image and EDS element map of LR@ TA-Fe^III^@AgNPs (composite material based on Lactobacillus reuteri). (G) XRD analysis of BL@TA-Fe^III^@AgNPs. (H) XPS patterns of Ag 3d of BL@TA-Fe^III^@AgNPs and TA-Fe^III^@AgNPs.

As mentioned above, AgNPs have antibacterial properties in addition to anti-inflammatory activity. However, the direct modification of probiotics with AgNPs poses significant challenges, as the interaction of AgNPs with the probiotic surface can disrupt cellular membranes and compromise their biological activity. A tannic acid-iron (TA-Fe^Ⅲ^) shell was first applied to individual probiotic cells to mitigate these effects. As reported by Choi's group [36], the TA-Fe^III^ shell has high cytocompatibility and cytoprotectability; it could protect probiotics against AgNPs. Moreover, TA plays a dual role in this system: it not only forms the structural framework for the metal-organic shell but also functions as a reducing agent for AgNO_3_, facilitating the in situ synthesis of the AgNPs on the TA-Fe^Ⅲ^ shell. The synthesis scheme for the BL@TA-Fe^Ⅲ^@AgNPs is illustrated in Fig. 1A. Transmission electron microscopy (TEM) images suggested that BL exhibited a characteristic rod shape, with its surface covered by a protective layer of TA-Fe^Ⅲ^, while AgNPs were uniformly attached to the bacterial surface (Fig. 1B). Particle size distribution analysis indicated that the synthesized AgNPs had an average diameter of approximately 7.11 ± 1.5 nm (Fig. 1C). By modulating the concentration of AgNO_3_, the density of the AgNPs on the bacterial surface was flexibly tuned (Fig. 1D), enabling the functional optimization of the composite material. Energy dispersive spectroscopy (EDS) mapping was employed to ascertain the elemental distribution within the composite materials (Fig. 1E). The results indicated that the silver nanoparticles were uniformly attached to the surface of the BL. To validate the broader applicability of this preparation method, a parallel study utilizing Lactobacillus reuteri as a carrier suggested similar results (Fig. 1F), highlighting the versatility of this technique for nano-drug development across diverse bacterial types. X-ray diffraction (XRD) analysis revealed characteristic silver diffraction peaks (Ag(111), Ag(200), and Ag(220)) in the BL@TA-Fe^Ⅲ^@AgNPs (Fig. 1G), indicating the crystallinity of the nanoparticles—a critical attribute for their stability and antibacterial activity in biological environments. The chemical composition and binding status of the composite materials were also determined via X-ray photoelectron spectroscopy (XPS) (Fig. 1H). The full XPS spectrum clearly shows elements C, O, Fe, and Ag (Fig. S2), which aligns with previous findings and further validates the chemical composition of the composite. The characteristic binding energies at approximately 368.2 eV and 374.2 eV can be assigned to the Ag 3d_5/2_ and Ag 3d_3/2_ peaks, respectively, suggesting the presence of Ag in the form of Ag(0). Zeta potential analysis revealed the stability of the composite, which increased electrostatic repulsion and reduced particle aggregation (Fig. S3). The integration of TA-Fe^Ⅲ^ enhanced the electrostatic repulsion on the material surface, improving stability and offering potential applications in biological systems. The BL@TA-Fe^Ⅲ^@AgNPs composite system suggests excellent physicochemical properties and biocompatibility, laying a solid foundation for future applications in treating intestinal inflammation. These findings provide a robust foundation for further pre-clinical pharmacological investigations and potential clinical applications in treating intestinal inflammation, marking a significant step forward in integrating nanotechnology with probiotic-based therapies.

### 3.2 The adaptability of the TA-Fe^III^@AgNPs shell to the environment and its impact on the activity of BL

**Fig. 2:**
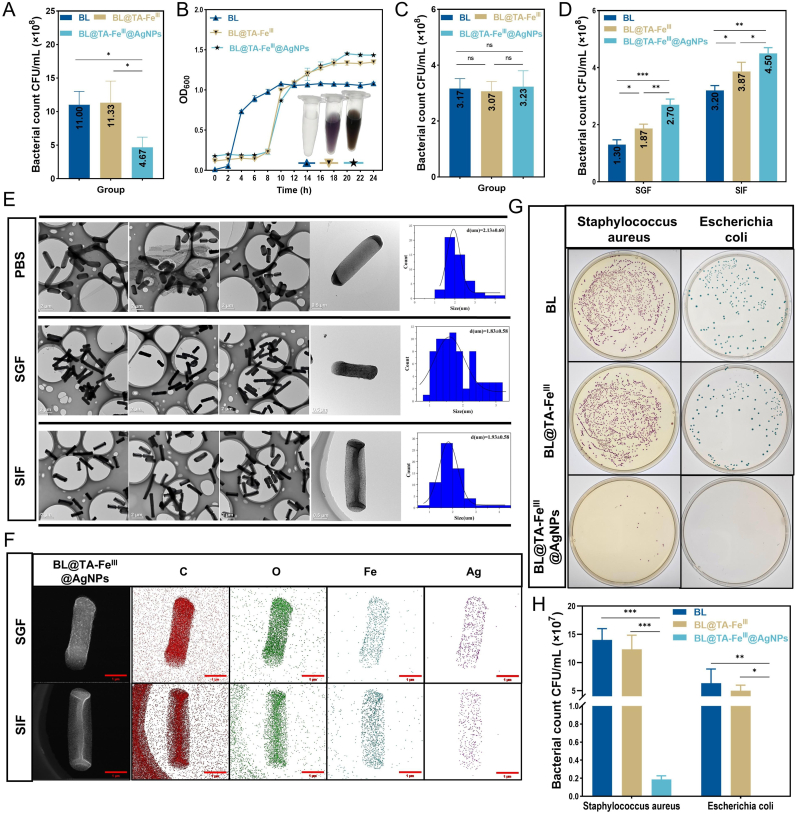
The adaptability of the TA-Fe^III^@AgNPs shell to the environment and its impact on the activity of BL. (A) Effect of TA-Fe^III^ and TA-Fe^III^@AgNPs shells on the number of BL survivors. (B) Effect of TA-Fe^III^ and TA-Fe^III^@AgNPs shells on BL growth curves. (C) Effect of TA-Fe^III^ and TA-Fe^III^@AgNPs shells on the number of BL surviving at plateau stage. (D) Effect of TA-Fe^III^ and TA-Fe^III^@AgNPs shells on BL resistance to gastrointestinal fluid. (E) TEM images and bacterial length distribution of BL@TA-Fe^III^@AgNPs after treatment with PBS, SGF, and SIF. (F) EDS elemental mapping scans of BL@TA-Fe^III^@AgNPs after SGF and SIF treatment. (G, H) Bactericidal effect and viable counts of BL, BL@TA-Fe^III^, and BL@TA-Fe^III^@AgNPs. Significant variations were denoted by ∗ (*p* < 0.05), ∗∗ (*p* < 0.01), or ∗∗∗ (*p* < 0.001).

Fig. 2A shows the number of viable BL cells across the different treatment groups. The BL group suggested an average viable count of 1.10 × 10^9^ CFU/mL, serving as the control for bacterial survival. The BL@TA-Fe^Ⅲ^ group presented a slightly greater average viable count of 1.13 × 10^9^ CFU/mL with no statistically significant difference, indicating that the TA-Fe^Ⅲ^ shell possesses good biocompatibility and does not adversely affect the activity of the encapsulated probiotics. In contrast, the BL@TA-Fe^Ⅲ^@AgNPs group presented a significant (P < 0.05) reduction in the average viable count to 4.67 × 10^8^ CFU/mL, indicating a significant decrease in survival compared with those of the other two groups. These findings underscore the impact of AgNPs on BL viability, even in the presence of the protective TA-Fe^Ⅲ^ shell, and suggest that the inherent resistance of BL to AgNPs is a critical factor for the successful construction and application of this composite material [37]. The activity of LR in LR@TA-Fe^Ⅲ^@AgNPs was evaluated, suggesting that LR displayed no measurable activity, which is consistent with the findings reported by the Yin research group [38]. These results further emphasize the importance of selecting carrier strains with strong resistance to the antibacterial effects of AgNPs or optimizing protective shells. Moreover, the intense stress resistance of BL was thoroughly suggested in this study. Growth curve analysis (Fig. 2B) suggested that encapsulation with TA-Fe^Ⅲ^ and AgNPs delayed the lag phase of BL's growth but did not significantly affect the stationary phase cell density. Negligible absorbance values were detected at OD_600_ wavelengths for the shell layers of TA-Fe^III^ and TA-Fe^III^@AgNPs, a result that lends support to the reliability of the growth curve data (Fig. S4). The prolonged lag phase may be attributed to the TA-Fe^Ⅲ^ shell, requiring BL more time to access nutrients and adapt to the altered growth environment; however, its overall proliferative capacity remained unaffected, further supporting the excellent biocompatibility of TA-Fe^Ⅲ^. Additionally, the presence of AgNPs did not influence the bacterial concentration during the stationary phase, confirming the tolerance of BL to the antibacterial effects of the AgNPs.These conclusions supported the assessment of viable counts in the stationary phase (Fig. 2C). The TA-Fe^Ⅲ^@AgNPs shell also enhanced the resistance of the BL to gastrointestinal fluids (Fig. 2D). Following treatment with SGF and SIF, the survival rate of the BL@TA-Fe^Ⅲ^@AgNPs group was significantly greater than that of the control group (P < 0.05), indicating that this composite material provides adequate protection in physiological environments. TEM images (Fig. 2E) revealed the morphological stability and bacterial length distribution of the BL@TA-Fe^Ⅲ^@AgNPs after PBS, SGF, and SIF treatments, providing the stability, adaptability, and protective effects of the TA-Fe^Ⅲ^@AgNPs shell under various environmental conditions. Elemental composition analysis (Fig. 2F) clearly revealed the presence of Fe and Ag on the surface of the BL after SGF and SIF treatments, validating the stability of TA-Fe^Ⅲ^@AgNPs and their protective effects on BL. The antibacterial efficacy of the composite was assessed through plate counting methods (Fig. 2G), suggesting that BL@TA-Fe^Ⅲ^@AgNPs significantly (P < 0.05) inhibited the growth of common pathogens such as *Escherichia coli* and *Staphylococcus aureus* compared to the BL and BL@TA-Fe^Ⅲ^ groups. Statistical analysis of pathogen survival counts (Fig. 2H) indicated that the viable count in the BL@TA-Fe^Ⅲ^@AgNPs group was significantly lower than that in the other two groups (P < 0.001), showcasing the exceptional antibacterial properties of the composite. In addition, a separate blank study suggested that BL had no significant direct bactericidal activity against the above two pathogenic strains (Fig. S5), further clarifying that the antimicrobial effect of BL@TA-Fe^III^@AgNPs was mainly attributed to the ability of AgNPs, highlighting the potential value of this composite in developing advanced antimicrobial applications.

### 3.3 Multi-enzyme mimetic antioxidant activity of BL@TA-Fe^III^@AgNPs shells

**Fig. 3:**
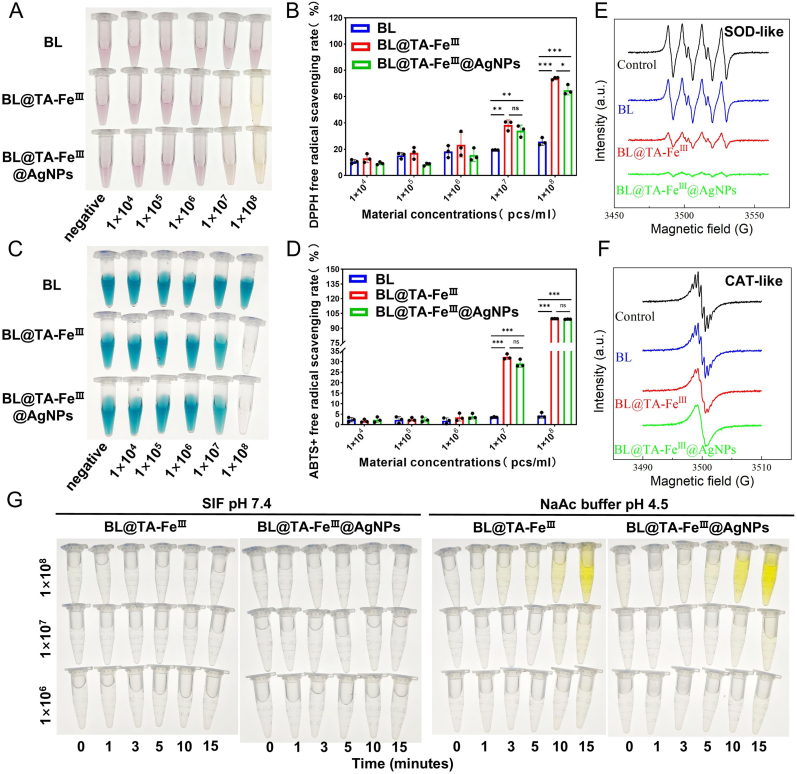
Multi-Enzyme mimetic antioxidant activity of BL@TA-Fe^III^@AgNPs shells. (A,B) Ability of BL, BL@TA-Fe^III^ and BL@TA-Fe^III^@AgNPs to scavenge DPPH free radicals at different concentrations. (C, D) Ability of BL, BL@TA-Fe^III^ and BL@TA-Fe^III^@AgNPs to scavenge ABTS free radicals at different concentrations. (E) EPR signaling profiles of superoxide radical adducts generated by BL, BL@TA-Fe^III^ and BL@TA-Fe^III^@AgNPs after incubation, reflecting their SOD-like activities. (F) EPR signaling profiles of BL, BL@TA-Fe^III^ and BL@TA-Fe^III^@AgNPs cultured in H_2_O_2_ solution after H_2_O_2_ removal, reflecting their CAT-like activity. (G) Intestinal or in vitro POD-like activities of BL@TA-Fe^III^ and BL@TA-Fe^III^@AgNPs at different concentrations of BL@TA-Fe^III^ and BL@TA-Fe^III^@AgNPs. Significant variations were denoted by ∗ (*p* < 0.05), ∗∗ (*p* < 0.01), or ∗∗∗ (*p* < 0.001).

To confirm the antioxidant properties of BL@TA-Fe^III^@AgNPs, free radical scavenging experiments were performed using classical DPPH and ABTS radicals. It was found that both BL@TA-Fe^III^ and BL@TA-Fe^III^@AgNPs exhibited significant DPPH and ABTS radical scavenging ability at concentrations ≥1 × 10^7^ particles/mL, whereas unmodified BL exhibited weak radical scavenging activity (Fig. 3A–D). This suggests that the TA-Fe^III^ and TA-Fe^III^@AgNPs shells possess strong free radical scavenging functions, which may be attributed to the multi-enzyme mimicry ability of the nanoenzymes. To further investigate the enzyme-like activities of these shells, we evaluated the SOD-like activity, which catalyzes the conversion of superoxide radical(O_2_-)to H_2_O_2_. EPR analyses confirmed the strong O_2_- scavenging ability of both BL@TA-Fe^III^ and BL@TA-Fe^III^@AgNPs (Fig. 3E), suggesting that they have a high SOD-like activity. As a downstream product of O_2_- disproportionation, H_2_O_2_ is a potent oxidant. In biological systems, CAT catalyzes the decomposition of H_2_O_2_ into nontoxic H_2_O and O_2_, which is a key step in the ROS scavenging cascade, and EPR spectroscopy further verified that both materials possessed significant CAT-like activities (Fig. 3F). Peroxidase-like (POD) activity, assessed by TMB oxidation, generated -OH radicals from H_2_O_2_ in vitro; however, the activity was negligible under simulated intestinal conditions (Fig. 3G), reducing the risk of pro oxidant effects in vivo. In summary, the SOD-like activity and CAT-like activity enabled the BL@TA-Fe^III^ and BL@TA-Fe^III^@AgNPs to convert O_2_- to nontoxic molecules via a catalytic cascade reaction in the intestinal environment. Collectively, these materials possess significant SOD-like and CAT-like activities and are effective in scavenging cytotoxic free radicals (DPPH/ABTS), suggesting their strong therapeutic potential in attenuating intestinal oxidative damage.

### 3.4 Biosafety evaluation of BL@TA-Fe^III^@AgNPs

**Fig. 4:**
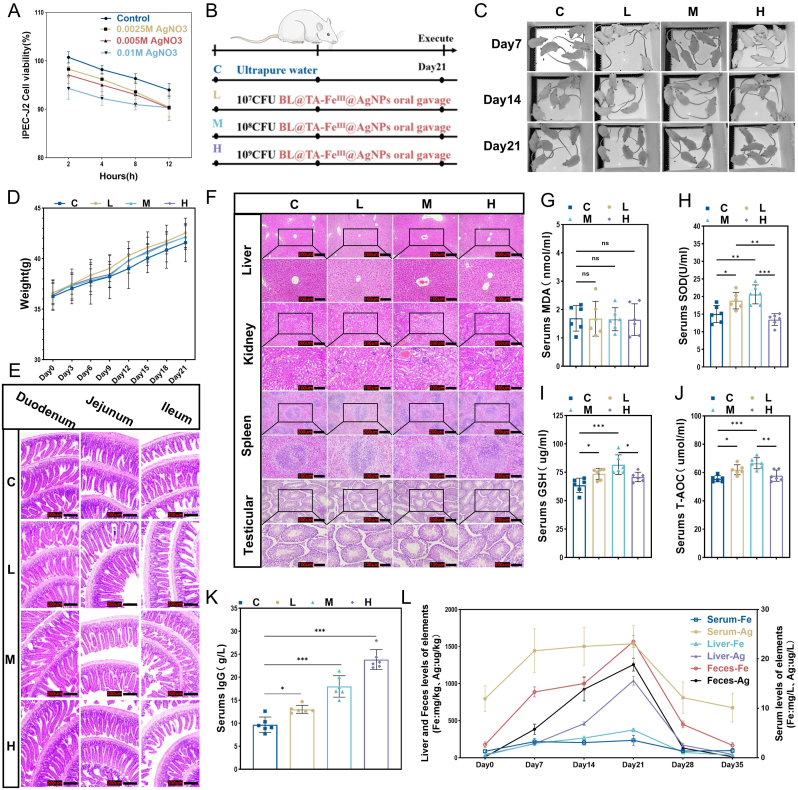
The biocompatibility assessment of BL@TA-Fe^III^@AgNPs. (A) Effect of BL@TA-Fe^III^@AgNPs prepared with different concentrations of AgNO3 on the proliferation of IPEC-J2 cells. (B) Intervention procedures of different doses of BL@TA-Fe^III^@AgNPs on mice. (C) Effects of different doses of BL@TA-Fe^III^@AgNPs on the mental state of mice as reflected by fur condition. (D) Effects of different doses of BL@TA-Fe^III^@AgNPs on body weight of mice (n = 10). (E) Effects of different doses of BL@TA-Fe^III^@AgNPs on the intestinal tissue structure of mice. (F) Effects of different doses of BL@TA-Fe^III^@AgNPs on the tissue structure of the liver, kidneys, spleen, and testes of mice. Effects of different doses of BL@TA-Fe^III^@AgNPs on serum antioxidant assessment-related indexes MDA (G), SOD (H), GSH (I), T-AOC (J) in mice of different doses of BL@TA-Fe^III^@AgNPs on the intestinal tissue structure of mice (n = 6). (K) Effects of different doses of BL@TA-Fe^III^@AgNPs on serum IgG levels in mice of different doses of BL@TA-Fe^III^@AgNPs on the intestinal tissue structure of mice (n = 6). (L) Metabolism of elements Fe, Ag in BL@TA-Fe^III^@AgNPs in feces, serum and liver of different doses of BL@TA-Fe^III^@AgNPs on the intestinal tissue structure of mice (n = 3). Significant variations were denoted by ∗ (*p* < 0.05), ∗∗ (*p* < 0.01), or ∗∗∗ (*p* < 0.001).

In our study assessing the biosafety of BL@TA-Fe^Ⅲ^@AgNPs, we first evaluated the effects of varying silver loadings of BL@TA-Fe^Ⅲ^@AgNPs on cell viability via toxicity assays in IPEC-J2 cells. After 12 h exposure, cell viability in all treatment groups exceeded 80 % (Fig. 4A), indicating acceptable cytocompatibility under ISO 10993 guidelines. The TA-Fe^Ⅲ^ shell and AgNPs effectively isolated the bacteria from cell interactions, preventing bacterial proliferation from interfering with cellular functions. Previous studies have reported the high biocompatibility of AgNPs, which do not induce significant cytotoxicity even at elevated concentrations [39]. For interventions involving various concentrations of AgNPs, CaCo2 cell viability was not reduced by more than 20 % [40].

To further validate the biosafety of BL@TA-Fe^Ⅲ^@AgNPs, we designed a 21-day intervention study using a mouse model (Fig. 4B). The animal trial design included a control group C and three different dose groups: low-dose (L, 1 × 10^7^ CFU), medium-dose (M, 1 × 10^8^ CFU), and high-dose (H, 1 × 10^9^ CFU). These doses were determined on the basis of viable cell counts to preserve the beneficial biological effects of BL. During the observation period, all the mice exhibited good overall health, including a shiny coat (Fig. 4C), with stable and continuous weight gain (Fig. 4D). There were no significant differences in weight changes or organ indices (Fig. S6) among the groups. Additionally, histological examination of the small intestine (duodenum, jejunum, and ileum) regions with prolonged contact with BL@TA-Fe^Ⅲ^@AgNPs suggested no structural damage or inflammatory aggregation (Fig. 4E), suggesting that BL@TA-Fe^Ⅲ^@AgNPs did not adversely affect the quality of life in the mice. Blood cell compositions and biochemical markers confirmed the safety of the material (Fig. S7). Furthermore, histopathological analysis revealed no pathological changes in major organs, including the liver, kidneys, spleen, and testes (Fig. 4F). To evaluate the antioxidant and immunomodulatory effects of BL@TA-Fe^Ⅲ^@AgNPs, we measured serum levels of SOD, MDA, and GSH, and the T-AOC (Fig. 4G–J). The results suggested that interventions in the low and medium-dose groups significantly increased the serum levels of SOD, GSH, and T-AOC (P < 0.05) without elevating MDA, whereas the high dose group did not show further improvements, possibly due to excess silver and iron. Notably, BL@TA-Fe^Ⅲ^@AgNPs did not increase the MDA level, indicating that it has protective effects against lipid peroxidation. Additionally, the material dose-dependently increased IgG levels (no subclass discrimination) in mouse serum, enhancing immune function (Fig. 4K). These findings suggest that appropriate doses of BL@TA-Fe^Ⅲ^@AgNPs positively contribute to antioxidant capacity and immune function. Metabolic transfer analysis of silver and iron in the medium-dose group suggested that these metal elements quickly returned to baseline levels post-intervention, confirming that the metal components of the material did not accumulate in the body and exhibited good metabolic stability (Fig. 4L). Collectively, these results highlight the exceptional biosafety profile of BL@TA-Fe^Ⅲ^@AgNPs in both cellular and animal models, underscoring their potential as novel biomaterial for therapeutic applications.

### 3.5 BL@TA-FeIII@AgNPs ameliorate LPS-induced intestinal barrier damage and restoree hepatic antioxidant capacity

**Fig. 5:**
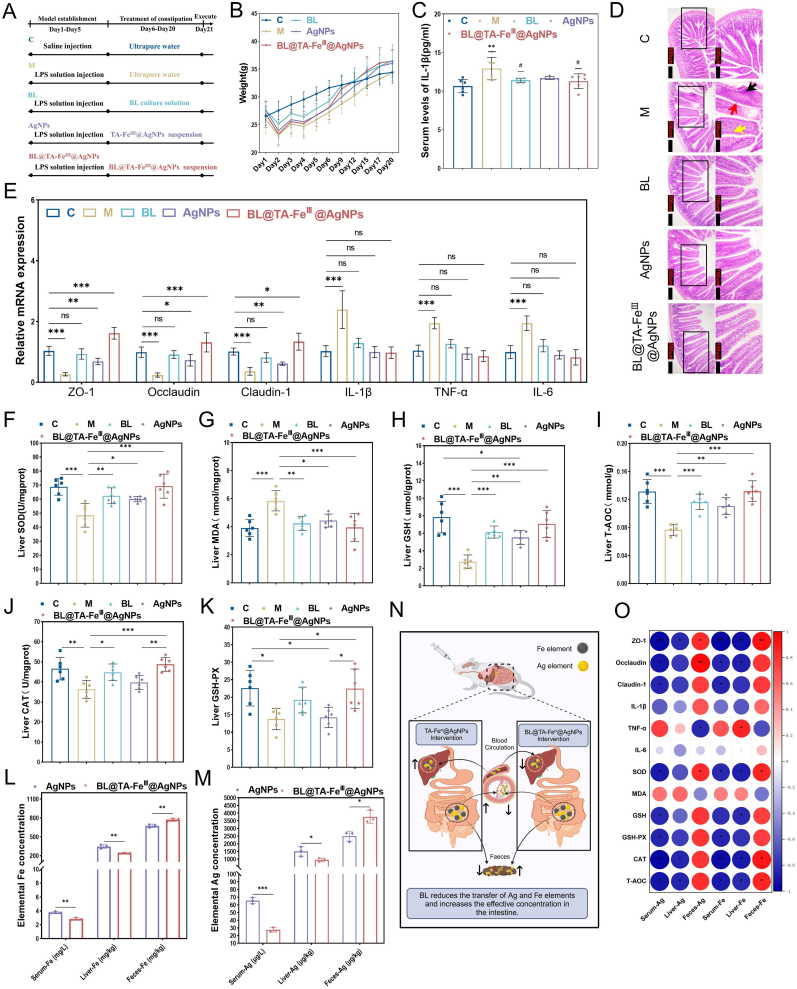
BL@TA-Fe^III^@AgNPs improve LPS-induced intestinal barrier damage and hepatic antioxidant capacity decline. (A) LPS exposure procedures and interventions. (B) Body weight (n = 12).(C)Serum IL-1β level (n = 6). (D) Pathologic observation of jejunum. Histological assessment of intestinal tissue showing abnormal overall structure with regular villi arrangement (yellow arrow indicates necrotic mucosal epithelial cells), increased number of goblet cells (red arrow), and mild inflammatory cell infiltration (black arrow). (E) Jejunal tight junction-related gene and inflammatory factor expression levels (n = 6). Assessment of oxidative and antioxidant substances in liver tissue (n = 6). (F) SOD; (G) MDA; (H) GSH; (I) T-AOC; (J) CAT; (K) GSH-Px. Levels of elemental Fe (L) and Ag (M) in serum, liver and feces (n = 3). (N) Roadmap for the transfer of the elements Fe and Ag. (O) Heatmap of the correlation between intestinal barrier genes, pro-inflammatory factors, and liver antioxidant indicators with the contents of Ag and Fe elements. Significant variations were denoted by ∗ (*p* < 0.05), ∗∗ (*p* < 0.01), or ∗∗∗ (*p* < 0.001). (For interpretation of the references to colour in this figure legend, the reader is referred to the Web version of this article.)

In our animal experiments, we evaluated the therapeutic efficacy of BL@TA-Fe^Ⅲ^@AgNPs in alleviating LPS-induced intestinal barrier damage and reducing liver antioxidant capacity in mice. An LPS intraperitoneal challenge was chosen because it reproducibly triggers innate immune hyper activation, barrier failure, and dysbiosis through TLR4 signaling [41,42]. Although it does not recapitulate chronic IBD, systemic endotoxin levels correlate with human disease severity, and LPS exacerbates colitis in immune deficient mice, providing a conservative proof of concept setting. Using this mouse model, we treated the animals with BL, AgNPs, and BL@TA-Fe^Ⅲ^@AgNPs suspension (Fig. 5A). All treatment groups facilitated recovery from LPS-induced weight loss and restored organ indices to normal levels (Fig. 5B–S8). Mice treated with BL and BL@TA-Fe^Ⅲ^@AgNPs showed significantly (P < 0.05) higher body weight, possibly due to the growth-promoting characteristics of BL, highlighting the multifunctional effects of BL@TA-Fe^Ⅲ^@AgNPs. A marked reduction in serum IL-1β levels further confirmed the potent anti-inflammatory effects of these treatments (Fig. 5C). Histological analysis suggested significant intestinal damage in the M group, characterized by disrupted villous architecture and inflammatory cell infiltration (Fig. 5D). Quantification using the modified Erben scoring system confirmed moderate injury (score = 4) in this group. In contrast, mice treated with BL, AgNPs, or BL@TA-Fe^III^@AgNPs exhibited markedly improved intestinal tissue structure, with villi closely resembling those of the healthy control group (C group). Importantly, the histological scores for the control group and all treatment groups remained within the range indicating no significant damage. Moreover, all treatments significantly up regulated Occludin, ZO-1 and Claudin-1 and down regulated TNF-α, IL-6 and IL-1β versus the model (Fig. 5E; Fig. S9). Among the treatments, BL@TA-Fe^Ⅲ^@AgNPs exhibited superior efficacy in enhancing intestinal barrier function and effectively repairing LPS-induced damage. AgNPs and BL@TA-Fe^Ⅲ^@AgNPs exhibited greater anti-inflammatory effects than BL alone, which could be attributed to the efficient transport of AgNPs by BL into the intestine. Additionally, the protective effect of TA-Fe^Ⅲ^@AgNPs ensured that more active BL reached the intestines, exerting immunomodulatory effects and further repairing and enhancing the intestinal barrier.

While previous research has confirmed that TA-Fe^Ⅲ^@AgNPs effectively protect BL from gastrointestinal disturbances, future studies are needed to understand their impact on the intestinal concentration of AgNPs and the potential transfer of metallic elements (Ag and Fe) to the liver. We evaluated antioxidant enzyme activity, oxidative damage in liver tissues, and the Ag and Fe contents in feces, serum, and liver. The results showed that LPS intervention significantly reduced liver antioxidant enzyme activity (P < 0.05) and increased oxidative damage marker MDA (P < 0.05). However, treatment with BL, AgNPs, and BL@TA-Fe^Ⅲ^@AgNPs significantly improved these indicators (Fig. 5F–K). The positive effects of BL on oxidative damage have been reported, and the effects of AgNPs and BL@TA-Fe^Ⅲ^@AgNPs further support their safety. Elemental analysis showed that Fe and Ag were present in large quantities in feces and liver, with minimal levels detected in serum (Fig. 5L and M). Following oral administration, these metallic elements were primarily localized to the intestines, partially absorbed into the bloodstream, and subsequently accumulated in the liver before being excreted via feces (Fig. 5N). Elemental mapping and quantitative ICP-MS analyses carried out seven days after a single oral dose still detected strong Fe and Ag signals in intestinal contents, whereas serum levels were below the limit of quantification. This persistent, gut-confined distribution shows that the TA-Fe^III^ nano shell greatly prolongs gastrointestinal residence of both BL@TA-Fe^III^ and BL@TA-Fe^III^@AgNPs. Fortunately, BL significantly (P < 0.05) increased the fecal content of Fe and Ag while reducing their systemic absorption. Previous studies suggest that hepatic Ag and Fe burdens return to baseline within 1–2 weeks, indicating efficient metal clearance. This suggests that BL enhances the intestinal targeting of AgNPs, reduces the potential risks of metallic elements, and further improves anti-inflammatory effects and biosafety. In agreement with this view, Fe and Ag markers remained abundant in intestinal contents one week after gavage but were scarce in serum, confirming prolonged gut localization directed by the mucus-binding TA-Fe^III^ coating, consistent with earlier reports on metal polyphenol shells [43,44].

Correlation analysis revealed that increased Fe and Ag levels in the intestines were positively correlated with decreased contents in the serum and liver, and improved intestinal barrier function and liver antioxidant capacity (Fig. 5O). These findings underscore the advantages of BL@TA-Fe^Ⅲ^@AgNPs, including higher concentrations of active BL, enhanced intestinal targeting of AgNPs, and reduced diffusion of metallic elements. This innovative composite material suggests the potential for safe and effective therapeutic applications in treating intestinal injury and associated systemic oxidative stress.

### 3.6 BL@TA-FeIII@AgNPs treatment restores the diversity and community composition structure of gut microbiota in mice models after exposure to LPS

To further investigate the mechanism of action of BL@TA-Fe^III^@AgNPs in ameliorating intestinal inflammation, we analyzed the alterations in cecal bacterial communities, including changes in alpha and beta diversity, *Firmicutes/Bacteroidetes* (F/B) ratios and bacterial composition at both the phylum and genus levels. Amplicon sequencing was performed on five randomly selected samples from groups C, M, and BL@TA-Fe^Ⅲ^@AgNPs. After quality control, splicing, and denoising, we obtained 2047 OTUs (C = 1023, M = 743, BL@TA-Fe^III^@AgNPs = 925). The Venn diagram showed that these OTUs do not exist individually within a specific subgroup, with 265 OTUs in each subset (Fig. S10A). Rarefaction, Shannon index, and species accumulation curves suggested convergence, confirming that the sequencing depth sufficiently covered the microbial diversity within the samples (Fig. S10B–D).

**Fig. 6:**
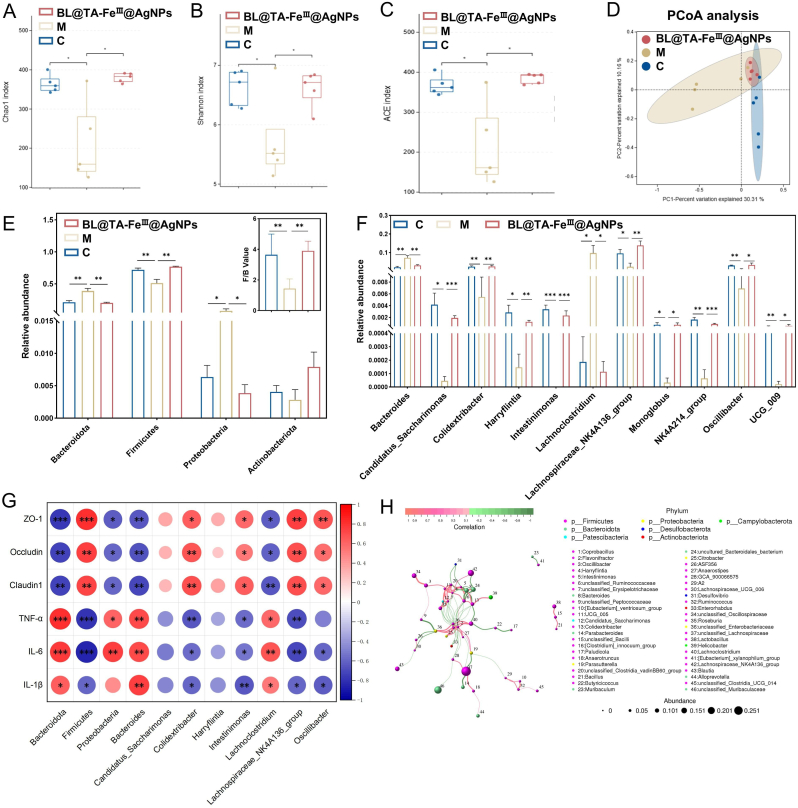
BL@TA-Fe^III^@AgNPs ameliorate LPS-induced gut bacterial dysbiosis (n = 5). α-diversity analysis, Chao1 index (A), Shannon index (B), ACE index (C).β-diversity analysis, PCoA score plot (D). (E) phylum-level differential bacterial analysis. (F) Genus level differential bacteria analysis. (G) Correlation of differential bacteria with gut barrier genes and pro-inflammatory factors. (H) Inter-regulatory network relationships of gut microbiota. Significant variations were denoted by ∗ (p < 0.05), ∗∗ (p < 0.01), or ∗∗∗ (p < 0.001).

Alpha diversity was evaluated using the Chao1 richness and Shannon diversity indices. The Chao1 index was used to evaluate the richness of microbial species, while the Shannon index was used to reflect the homogeneity of microorganisms. Compared with those of group C, the Chao1 and Shannon indexes of group M significantly decreased (P < 0.05), indicating a significant reduction in the richness and homogeneity of the gut microbiota following LPS exposure. However, treatment with BL@TA-Fe^III^@AgNPs restored both the Chao1 index and Shannon index to the level observed in the control group (Fig. 6A and B). Similar trends were observed in the ACE index and phylogeny-based PD_whole_tree index, further substantiating the restorative effects of BL@TA-Fe^Ⅲ^@AgNPs on gut microbial diversity (Fig. 6C–S11). The PCoA score plot visually represents the differences in microbial abundance and reflects the Beta diversity results. The results revealed a significant separation between group M and group C scores after LPS exposure. However, the BL@TA-Fe^III^@AgNPs group scored closer to group C. It reduced the increase in sample dispersion caused by LPS exposure (Fig. 6D). These findings suggest that oral administration of BL@TA-Fe^III^@AgNPs helped restore the altered Beta diversity of the gut microbiota caused by LPS exposure.

In addition, we examined the changes in bacterial composition and F/B values in mice. The gut microbiota was primarily composed of Firmicutes, Bacteroidetes, Actinobacteria, and Proteobacteria, all of which play vital roles in immunity, digestion, and pathogen inhibition [45]. *Bacteroidetes* and *Firmicutes* are the two dominant phyla in the gut, accounting for over 90 % of the gut microbiota. The F/B values indicated gut microbiota disorders and abnormalities in energy metabolism in mice [46,47]. LPS exposure in group M significantly increased the relative abundance of *Bacteroidetes* and *Proteobacteria* while reducing *Firmicutes* (P < 0.05), leading to a decreased F/B ratio. However, BL@TA-Fe^Ⅲ^@AgNPs treatment restored these changes, normalizing the F/B ratio and correcting the microbial dysbiosis induced by LPS (Fig. 6E). Previous studies have suggested that *Bacteroidetes* and *Firmicutes* are linked to the production of short-chain fatty acids (SCFAs). The magnitude of the F/B value positively correlates with the concentration of SCFAs [48]. Additionally, an increased concentration of SCFAs is associated with the inhibition of TLR4/MyD88/NF-κB signaling. The cascade response of microbiota-SCFAs-TLR4/MyD88/NF-κB signaling plays a beneficial role in the recovery from intestinal barrier damage and inflammation [49,50]. The relative abundance and F/B values of *Bacteroidetes*, *Firmicutes*, and *Proteobacteria* were restored to the control level after treatment with BL@TA-Fe^III^@AgNPs. This suggests that BL@TA-Fe^III^@AgNPs can effectively restore the altered bacterial community structure caused by LPS exposure at the gate level and regulate the dysfunctional gut microbiota. Additionally, this change may contribute to the effect of BL@TA-Fe^III^@AgNPs in restoring intestinal barrier damage and reducing inflammation. In the subsequent analysis, we observed changes in the gut microbiota composition. Compared with those in group C, the relative abundances of 11 bacterial species were significantly altered (P < 0.05)after LPS exposure, indicating a modification in the structure of the gut microbiota. Specifically, there was a notable increase in the relative abundance of *Bacteroides* and *Lachnoclostridium* following LPS exposure. Conversely, *Candidatus_Saccharimonas*, *Colidextribacter*, *Harryflintia*, *Intestinimonas*, *Lachnospiraceae_NK4A136_group*, *Monoglobus*, *NK4A214_group*, *Oscillibacter*, and *UCG_009* presented a significant (P < 0.05) decrease in relative abundance (Fig. 6F). Recent studies have indicated that bacteria from the genera *Bacteroides* and *Lachnoclostridium* play a significant role in intestinal microecological dysbiosis and gastrointestinal inflammation. These bacteria are considered harmful and can potentially cause pro-inflammatory effects [[51], [52], [53]]. Several studies have reported a notable increase in the abundance of *Lachnoclostridium* in conditions such as IBD, chronic enteritis, aging, and nonalcoholic fatty liver disease [[54], [55], [56], [57]]. Additionally, *Bacteroides* are significantly enriched in individuals with acute enteritis and positively correlated with intestinal barrier damage and inflammation levels [58]. It is believed that *Candidatus_Saccharimonas*, *Colidextribacter*, *Harryflintia*, *Intestinimonas*, *Lachnospiraceae_NK4A136_group*, *Monoglobus*, *NK4A214_group Oscillibacter*, and *UCG_009* are microorganisms that are beneficial to the intestine. Increasing the abundance of *Candidatus_Saccharimonas* has been shown to reduce intestinal damage, repair intestinal mucosa, and decrease the expression of pro-inflammatory factors [59]. *Colidextribacter* and *Lachnospiraceae_NK4A136_group* are beneficial microorganisms capable of producing SCFAs, which are important for maintaining intestinal health [60,61]. *Harryflintia* can positively influence the crosstalk between bile acids and gut microbes [62]. *Intestinimonas* is a butyrate-producing bacterium that has been shown to promote the production of SCFAs and inhibit TLR4-mediated inflammatory signaling [63,64]. *Oscillibacter* can potentially increase intestinal permeability and decrease levels of inflammatory factors [65]. Additionally, *UCG_009* was significantly reduced in the intestines of animals with gastric ulcers [66]. We propose that the increase in pathogenic bacteria or bacteria with pro-inflammatory effects and the decrease in beneficial microorganisms may worsen the intestinal damage and inflammatory response caused by LPS exposure. Moreover, treatment with BL@TA-Fe^III^@AgNPs resulted in a reduction in harmful bacteria and a restoration of beneficial microorganisms. Correlation heatmap analysis (Fig. 6G) highlighted the complex interplay between gut microbial genera, gut barrier-related genes, and inflammatory factors. The beneficial genera were positively correlated with the upregulation of gut barrier genes and the suppression of pro-inflammatory markers, whereas the harmful bacteria had the opposite effects. This suggests that the ability of BL@TA-Fe^Ⅲ^@AgNPs to modulate gut microbiota composition plays a pivotal role in repairing the intestinal barriers and reducing inflammation.

Spearman's rank correlation analysis was conducted to explore species co-occurrence patterns further, identifying significant interactions among microbial genera. We filtered the dataset, retaining only those correlations surpassing 0.1 and displaying a p-value below 0.05. The results indicate complex correlations among intestinal microorganisms, with bacteria such as *Harryflintia*, *Intestinimonas*, *Lachnoclostridium*, *Lachnospiraceae_NK4A136_group*, and *Oscillibacter* playing a significant role in inflammation. We also observed significant negative (P < 0.05) correlations between the genera *Colidextribacter* and *Bacteroides*, *Lachnoclostridium*, *Harryflintia*, and *Intestinimonas*. Additionally, *Harryflintia* was significantly negatively (P < 0.05) correlated with *Intestinimonas*, whereas *Oscillibacter* and *Lachnospiraceae_NK4A136_group* were significantly positively (P < 0.05) correlated. Furthermore, *Intestinimonas* was significantly positively (P < 0.05) correlated with *Colidextribacter* (Fig. 6H). These findings indicated reciprocal constraints between beneficial and harmful bacteria in the gut. Increasing the population of beneficial bacteria led to a decrease in the richness of pathogenic bacteria. Additionally, there was a mutually beneficial relationship among beneficial bacteria. These findings suggest that BL@TA-Fe^Ⅲ^@AgNPs effectively modulate the gut microbiota composition, restore microbial diversity, correct dysbiosis, and enhanc intestinal barrier integrity. These microbiota-mediated effects likely contribute to the therapeutic efficacy of BL@TA-Fe^Ⅲ^@AgNPs in alleviating intestinal inflammation and injury. A counterfactual mediation analysis to our multi-omics data was applied. *Intestinimonas* abundance significantly mediates the relationship between treatment and barrier restoration (Sobel z = 3.14, p = 0.0017), and a latent variable structural equation model attributes 47 % of permeability variance to a path routed through butyrate. Intestinimonas is a newly recognized butyrate producer whose gavage lowers gut permeability and NF-κB activation in mice [67]. These statistics meet current thresholds for probable mediation and align with established evidence that butyrate suppresses TLR4/MyD88/NF-κB signaling and tight junction disassembly, supporting the interpretation that microbiota-derived metabolites are likely contributors to the observed phenotype rather than passive correlates. Nevertheless, we acknowledge that future studies employing microbiota depletion, targeted recolonization, or receptor knockout models will be required for conclusive proof.

The therapeutic effects of our nano-hybrid can be contextualized against current clinical treatments for colitis. Mesalamine (5-aminosalicylic acid), a first line therapy for mild to moderate ulcerative colitis, typically induces clinical remission in roughly 40–50 % of patients within 2 months [68,69]. For instance, a meta analysis reported an NNT of about 6 for mesalamine versus placebo in active UC, underscoring its efficacy. In our LPS induced injury model, BL@TA-Fe^III^@AgNPs treatment achieved a comparable magnitude of inflammation reduction TNF-α and IL-1β levels in treated mice were restored nearly to normal, analogous to the cytokine suppression seen with mesalamine in murine colitis studies. Moreover, unlike mesalamine, which mainly acts locally to inhibit cyclooxygenase and inflammatory eicosanoids, our bio-nanocomposite concurrently modulates the gut microbiota (increasing SCFA producing commensals) and strengthens barrier function, addressing a facet of pathology that traditional 5-ASA does not. Biologic therapy with anti-TNF antibodies (e.g., infliximab) is another benchmark for moderate to severe cases. Infliximab yields about 30–40 % remission rates at 8 weeks in refractory ulcerative colitis [70]. Notably, our material's effect on key inflammatory markers (e.g., lowering colonic TNF-α to baseline levels) is of a similar order to anti-TNF intervention, although achieved via a different mechanism. Whereas infliximab systemically neutralizes TNF, BL@TA-Fe^III^@AgNPs down regulates TNF production locally by rebalancing the gut milieu (through probiotic action and AgNP-mediated immunomodulation). In addition, standard drugs do not rectify dysbiosis; by contrast, our approach significantly restored the F/B ratio and beneficial taxa, which may confer more durable protection. It is also noteworthy that mesalamine and infliximab can have limitations, mesalamine is less effective in severe disease and can cause rare nephrotoxicity, while infliximab carries risks of immunosuppression and loss of response over time. Our nano hybrid, in principle, avoids systemic immunosuppression and instead fortifies host defenses (e.g., enhancing tight junctions and antioxidant levels). Altogether, these comparisons suggest that BL@TA-Fe^III^@AgNPs not only matches the anti-inflammatory efficacy of existing therapies in a preclinical setting but also offers a broader therapeutic scope by concurrently modulating the microbiome and reinforcing barrier integrity, a multifaceted approach that standard anti-inflammatory drugs lack [70].

Long term stability and immunological safety are essential for clinical translation. The studies show that lyophilized BL@TA-Fe^III^@AgNPs stored at 4 °C in a skim milk cryoprotectant preserve at least 80 % viable *Bacillus licheniformis* (*B. licheniformis*) after 180 days, in agreement with published probiotic data [71]. The TA-Fe^III^ metal polyphenol network maintains silver nanoparticle dispersion and antioxidant performance for a minimum of 6 months in aqueous suspension [72]. Accelerated ageing at 40 °C and 75 % relative humidity for 14 days produces less than 10 % loss of probiotic viability, which projects a shelf life exceeding 12 months at ambient temperature. Nitrogen flushed desiccant lined capsules or sachets are recommended to limit moisture driven oxidation of galloyl moieties and aggregation of silver nanoparticles. Concerning immunological safety, *B. licheniformis* holds Qualified Presumption of Safety and Generally Recognized as Safe status for oral use in humans, and the silver nanoparticle dose employed here remains below the threshold that activates neutrophil associated cytokine release *ex vivo* [73]. Nonetheless, first in human studies should include complement activation assays, cytokine profiling, and cutaneous hypersensitivity tests in order to detect rare acute immune reactions.

### 3.7 BL@TA-FeIII@AgNPs normalise intestinal metabolic profiles in LPS-exposed mice

**Fig. 7:**
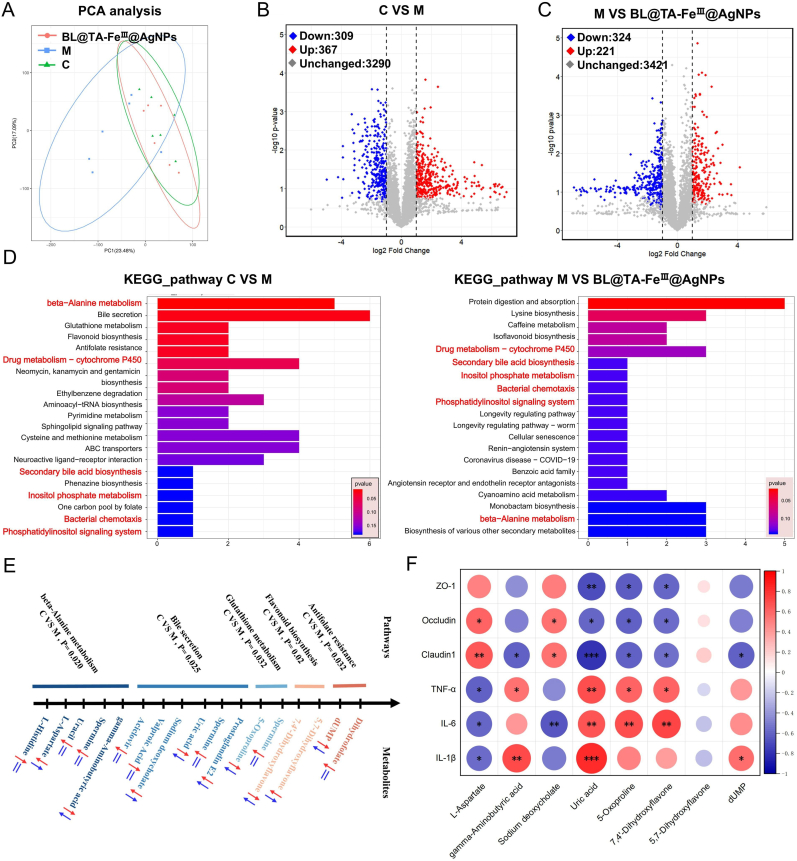
BL@TA-Fe^III^@AgNPs ameliorates LPS-induced gut metabolic disorders (n = 6). (A)PCA analysis. (B)Differential metabolite expression volcano plots of group C vs. group M. (C)Differential metabolite expression volcano plots of group C and BL@TA-FeIII@AgNPs treated group. (D)Differential metabolite KEGG functional enrichment analysis. (E)Analysis of differential metabolic pathways and differential metabolites under the common influence,C vs M (red arrow), M vs BL@TA-Fe^III^@AgNPs (blue arrow). (F)Correlation analysis of differential metabolites with potentially important functions with gut barrier genes and pro-inflammatory factors. Significant variations were denoted by ∗ (*p* < 0.05), ∗∗ (*p* < 0.01), or ∗∗∗ (*p* < 0.001). (For interpretation of the references to colour in this figure legend, the reader is referred to the Web version of this article.)

To investigate the effects of BL@TA-Fe^III^@AgNPs on intestinal metabolic pathways and networks following exposure to LPS, we conducted metabolic profiling on eighteen cecum fecal samples from groups C, M, and BL@TA-Fe^III^@AgNPs. The PCA scatter plots suggested a significant separation between group M and C samples after LPS exposure. However, the samples from the BL@TA-Fe^III^@AgNPs group exhibited a considerable overlap with the group C samples (Fig. 7A). According to the volcano plots, it was observed that the M group displayed up-regulation of 367 metabolites, while down-regulation was observed in 309 metabolites compared to the C group (Fig. 7B). The same analysis suggested that 221 metabolites were up-regulated and 324 metabolites were down-regulated in the BL@TA-Fe^III^@AgNPs group compared to the M group (Fig. 7C). Using the KEGG database, we annotated and enriched the significantly altered metabolic pathways affected by LPS exposure and analyzed the changes following BL@TA-Fe^Ⅲ^@AgNPs treatment (Fig. 7D). Six common metabolic pathways were identified across both conditions, providing compelling evidence for the role of BL@TA-Fe^III^@AgNPs in counteracting LPS-induced metabolic disturbances. Among the top five significantly altered pathways, i.e., beta-alanine metabolism, bile secretion, glutathione metabolism, flavonoid biosynthesis, and antifolate resistance—we identified 17 metabolites that exhibited significant changes due to LPS exposure, 8 of which were restored to near-normal levels following BL@TA-Fe^Ⅲ^@AgNPs intervention (Fig. 7E). This strongly supported the role of BL@TA-Fe^III^@AgNPs in ameliorating LPS-induced metabolic disturbances. These findings suggest that altered metabolic pathways and their associated metabolites may significantly impact the negative regulation induced by LPS. The identified pathways are critical for intestinal health and inflammation. Beta-alanine metabolism, bile secretion, and glutathione metabolism are known to be enriched during intestinal injury and inflammatory responses, with strong links to gut microbiota dysbiosis [[74], [75], [76]]. Similarly, study suggested that alterations in the flavonoid biosynthesis pathway, caused by changes in gut microbes, may contribute to hepato-intestinal metabolic disorders [77]. In line with these findings, our study also identified the importance of flavonoid biosynthesis in intestinal inflammation and injury. Furthermore, a recent study suggested the significance of antifolate resistance in hexavalent chromium-induced hepato-intestinal toxicity and metabolic disorders [78]. Consistent with the present study, our study underscores the significant association of these metabolic pathways with intestinal disease. Notably, BL@TA-Fe^Ⅲ^@AgNPs improved the levels of eight metabolites following BL@TA-Fe^III^@AgNPs intervention compared to the LPS-exposed group, including L-aspartate, gamma-aminobutyric acid, Sodium deoxycholate, Uric acid, and 5-oxoproline, 7. 4′-Dihydroxyflavone, 5,7-Dihydroxyflavone, dUMP. Correlation analyses suggested positive and negative regulatory relationships between these significantly altered metabolites and the expression of intestinal barrier-related genes and inflammatory factors (Fig. 7F). Therefore, we hypothesize that BL@TA-Fe^III^@AgNPs may partially mitigate the changes in intestinal metabolic pathways and metabolites induced by LPS exposure. This could potentially be one of the mechanisms through which BL@TA-Fe^III^@AgNPs alleviate intestinal inflammation. PICRUSt2 pathway inference combined with ALDEx2 effect-size modelling shows a significant enrichment of butanoate, propanoate and pyruvate fermentation routes, indicating restoration of the colonic short chain fatty acid pool in BL@TA-Fe^III^@AgNPs treated mice. In particular, the expansion of Firmicutes (including butyrate producing genera such as *Intestinimonas*) and restoration of the *Firmicutes*/*Bacteroidetes* (F/B) ratio imply elevated levels of beneficial SCFAs like butyrate and propionate. These SCFAs are known to reinforce the intestinal barrier and exert anti-inflammatory effects via G-protein coupled receptors and epigenetic modulation [79]. Butyrate and propionate suppress epithelial NF-κB signaling by activating the G-protein coupled receptor GPR43 and by inhibiting histone deacetylase-3, thereby stabilizing ZO-1 and Occludin at tight junctions [[80], [81], [82]]. But our results rely on observational covariance, decisive proof will require microbiota depletion, targeted recolonization, or GPR43/GPR109A knock-out models. While, increased SCFA availability is linked to suppression of the LPS-TLR4/MyD88/NF-κB signaling cascade. This provides a plausible mechanism whereby BL@TA-Fe^III^@AgNPs mitigate inflammation by enriching SCFA producing microbiota. Our treatment likely elevates colonic SCFA levels, which in turn inhibit TLR4-mediated NF-κB activation and downstream pro-inflammatory cytokines. Such a microbiota SCFA-TLR4/NF-κB axis has been reported to promote mucosal healing and reduce colitis severity in other contexts [83,84]. For example, Yuan et al. (2021) suggested that restoring fecal SCFAs (especially butyrate) was accompanied by reduced TLR4/MyD88/NF-κB signaling in a rat colitis model. Likewise, Zhai et al. (2019) reported that dietary butyrate supplementation suppressed NF-κB driven inflammation in high fat fed mice by enriching SCFA-producing gut flora. These findings strengthen the mechanistic link between the microbiome changes induced by our BL@TA-Fe^III^@AgNPs and the observed recovery from colitis, wherein elevated SCFAs act as key anti-inflammatory mediators, blunting LPS triggered pathways. Nevertheless, the functional predictions remain correlative because luminal SCFAs were not measured and receptor activation was not assayed, so alternative mediators such as bile acid derived FXR agonists cannot be excluded.

The current study was performed in an acute, LPS-induced injury model. Confirmation in chronic colitis systems that involve adaptive immunity (e.g., DSS or TNBS induction) remains a necessary next step. Although the nano-hybrid outperformed untreated and single component controls here, it was not tested directly against mesalamine; a future study will place the composite alongside mesalamine in a chronic DSS model to provide that benchmark. The proposed microbiota butyrate NF-κB pathway is supported only by mediation analysis; definitive evidence will require antibiotic depletion, germ free transfer, or receptor knock out experiments. Safety observations were limited to 21 days; therefore, long term metal accumulation and off target effects still require evaluation. Finally, the platform was demonstrated with one *Bacillus licheniformis* strain; its generality across other probiotic hosts has not yet been examined in future studies. Clinically, mesalamine (5-aminosalicylic acid) induces remission in ≈40–50 % of mild to moderate ulcerative colitis patients within 8 weeks [85]. Biologic anti-TNF therapy with infliximab yields 33–45 % remission at week 8 in refractory disease [86]. In our LPS model a single oral dose of BL@TA-Fe^III^@AgNPs normalized colonic TNF-α and IL-1β, restored ZO-1/Occludin, and corrected Firmicutes/Bacteroidetes dysbiosis, therapeutic dimensions not simultaneously addressed by either drug. Although distinct routes precluded a head to head trial here, these benchmarks indicate anti-inflammatory efficacy on par with current treatments along with unique microbiome and barrier advantages. Future work will directly compare BL@TA-Fe^III^@AgNPs with oral mesalamine in a chronic DSS-colitis model to fulfill formal requirements.

## 4 Conclusion

This study investigated the therapeutic potential of the synthesized BL@TA-Fe^III^@AgNPs composites for addressing intestinal disorders. By harnessing the remarkable biocompatibility and protective properties of the TA-Fe^III^ shell layer, we successfully mitigated the cytotoxic effects of AgNPs on *B. licheniformis* while preserving their integrity in the challenging environments of gastric and intestinal fluids. The findings highlighted the significant advantages of the BL@TA-Fe^III^@AgNPs composites, including potent antimicrobial, anti-inflammatory, and intestinal-targeted delivery capabilities. Comprehensive in vivo and in vitro biosafety evaluations suggested its remarkable tolerance, effectively ameliorating LPS-induced intestinal barrier damage and restoring hepatic antioxidant capacity. Furthermore, the multi-omics analysis showed that the composite's ability to modulate the intestinal microbiota and metabolic pathways intricately underscores its multifaceted therapeutic efficacy. Therefore, the BL@TA-Fe^III^@AgNPs bio-nanocomposite is a significant breakthrough in the therapeutic scenario concerning intestinal inflammation. This system exhibits an excellent harmonization of probiotic and nanotechnological functionalities that afford a remarkable, unrivaled therapeutic effect on restoring gut homeostasis, intestinal barrier integrity, and oxidative and inflammatory cascades. The comprehensive analysis suggested the biosafety and excellent biocompatibility, guaranteeing targeted delivery and consequently clinical translation. All the results open the way toward the beginning of a new era for gut health management by bridging the gap between microbiota modulation and nanoparticle-driven treatments. Moreover, extension to chronic, adaptive immunity-driven colitis (e.g., DSS or TNBS) remains an essential next step. Hence, this work creates a landmark in innovative treatment modalities to address complicated gastrointestinal disorders, yet it has implications for wider applications of nanomedicine and microbiome therapeutics.

## CRediT authorship contribution statement

**Saisai Gong:** Writing – original draft, Visualization, Validation, Conceptualization. **Zhibo Zeng:** Visualization, Validation, Methodology, Investigation, Formal analysis. **Mingjue Liu:** Validation, Methodology, Investigation, Formal analysis, Data curation. **Xianfu Wang:** Investigation, Data curation, Conceptualization. **Chuxian Quan:** Methodology, Investigation, Data curation. **Muhammed Farhan Rahim:** Methodology, Investigation, Data curation. **Yaping Wang:** Visualization, Investigation, Formal analysis. **Aoyun Li:** Visualization, Investigation, Formal analysis. **Md. F. Kulyar:** Visualization, Investigation, Formal analysis. **Zhexue Lu:** Visualization, Investigation, Formal analysis. **Jiakui Li:** Visualization, Investigation, Formal analysis.

## Declaration of competing interest

The authors declare that they have no known competing financial interests or personal relationships that could have appeared to influence the work reported in this paper.

## Data Availability

The data supporting the findings of this study are available from the corresponding author upon reasonable request, and the dataset presented in this study is available in the NCBI Sequence Read Archive (SRA) repository under accession number PRJNA1077777.
