# Supplementary material for: Tannic acid-iron stabilized probiotic silver nano hybrids: Multi-target gut microbiota modulation and intestinal barrier restoration
Source: Mater Today Bio. 2025 Jul 16;33:102106. doi: 10.1016/j.mtbio.2025.102106 (PMC12302189; doi:10.1016/j.mtbio.2025.102106)
Supplement: Multimedia component 1 [file mmc1.docx]

**Supporting Information**

**Tannic Acid-Iron Stabilized Probiotic-Silver Nano-Hybrids: Multi-Target Gut Microbiota Modulation and Intestinal Barrier Restoration**

*Saisai Gong^1†^, Zhibo Zeng^1†^,* *Mingjue Liu^1^, Xianfu Wang^2^, Chuxian Quan^1^, Muhammed Farhan Rahim^1^, Yaping Wang^5^, Aoyun Li^4^, Md. F. Kulyar^1*^,Zhexue Lu^2*^, Jiakui Li^1,3*^*

^1^College of Veterinary Medicine, Huazhong Agricultural University, Wuhan, China

^2^College of Chemistry, Huazhong Agricultural University, Wuhan, China
^3^College of Animals Husbandry and Veterinary Medicine, Tibet Agriculture and Animal Husbandry University, Linzhi, China
^4^College of Veterinary Medicine, Henan Agricultural University, Zhengzhou, China
^5^College of Animal Science and Technology, Henan Agricultural University, Zhengzhou, China

*Correspondence

**Zhexue Lu -** College of Science, Huazhong Agricultural University, Wuhan, China; ORCID: 0000-0002-6083-5443; Email: [zhexuelu@mail.hzau.edu.cn](mailto:zhexuelu@mail.hzau.edu.cn)

**Jiakui Li -** College of Veterinary Medicine, Huazhong Agricultural University, Wuhan, China; College of Animals Husbandry and Veterinary Medicine, Tibet Agriculture and Animal Husbandry University, Linzhi, Tibet, People’s Republic of China;orcid.org/0000-0002-6065-6648;Email: lijk210@mail.hzau.edu.cn

**Table S1.** Intestinal injury scoring scale.

| **Parameters** | **Scoring Criteria** |
| --- | --- |
| Epithelial lesions | 0=intact, 1=localized detachment, 2=focal necrosis, 3=extensive necrosis |
| Inflammatory infiltration | 0=absent, 1=mild mucosal layer, 2=moderate, 3=severe + crypt abscesses |
| Deformation of villous structure | 0=normal, 1=mild shortening, 2=moderate disruption, 3=villi loss |
| Decreased number of cup cells | 0=normal, 1=mildly reduced, 2=significantly reduced, 3=absent |

**Table S2.** Target gene and internal reference gene primer synthesis sequences.

| Gene Name | Sequence (5' to 3') |
| --- | --- |
| β-actin-F | TGCTGTCCCTGTATGCCTCTG |
| β-actin-R | TGATGTCACGCACGATTTCC |
| ZO-1-F | AACCCGAAACTGATGCTGTGGATAG |
| ZO-1-R | CGCCCTTGGAATGTATGTGGAGAG |
| Occludin-F | GGCCTTTTGAAAGTCCACCTC |
| Occludin-R | AGGCAAATATGGCGATGCAC |
| Claudin 1-F | GCTGGGTTTCATCCTGGCTTCTC |
| Claudin 1-R | CCTGAGCGGTCACGATGTTGTC |
| TNF-α-F | CGCTCTTCTGTCTACTGAACTTCGG |
| TNF-α-R | GTGGTTTGTGAGTGTGAGGGTCTG |
| IL-6-F | TAGTCCTTCCTACCCCAATTTCC |
| IL-6-R | TTGGTCCTTAGCCACTCCTTC |
| IL-1β-F | CACTACAGGCTCCGAGATGAACAAC |
| IL-1β-R | TGTCGTTGCTTGGTTCTCCTTGTAC |

**Table S3.** Antibody information.

| Antibody Name | Production Company | Catalog Number |
| --- | --- | --- |
| β-actin Rabbit mAb(High Dilution) | ABclonal Technology Co.,Ltd. | AC026 |
| ZO-1 Rabbit Polyclonal Antibody | Proteintech Group, Inc. | 21773-1-AP |
| Occludin Rabbit Polyclonal Antibody | Proteintech Group, Inc. | 27260-1-Ap |
| Claudin 1 Rabbit Polyclonal Antibody | Proteintech Group, Inc. | 13050-1-AP |
| TNF-α Rabbit pAb | ABclonal Technology Co.,Ltd. | A0277 |
| IL-6 Polyclonal Antibody | Taizhou Baijia Biotechnology Co., Ltd. | IPB0062 |
| IL1β Rabbit pAb | ABclonal Technology Co.,Ltd. | A11369 |
| HRP-conjugated Affinipure Gota Anti-Rabbit IgG(H+L) | Proteintech Group, Inc. | SA00001-2 |

**Fig. S1** **BL growth curve.**


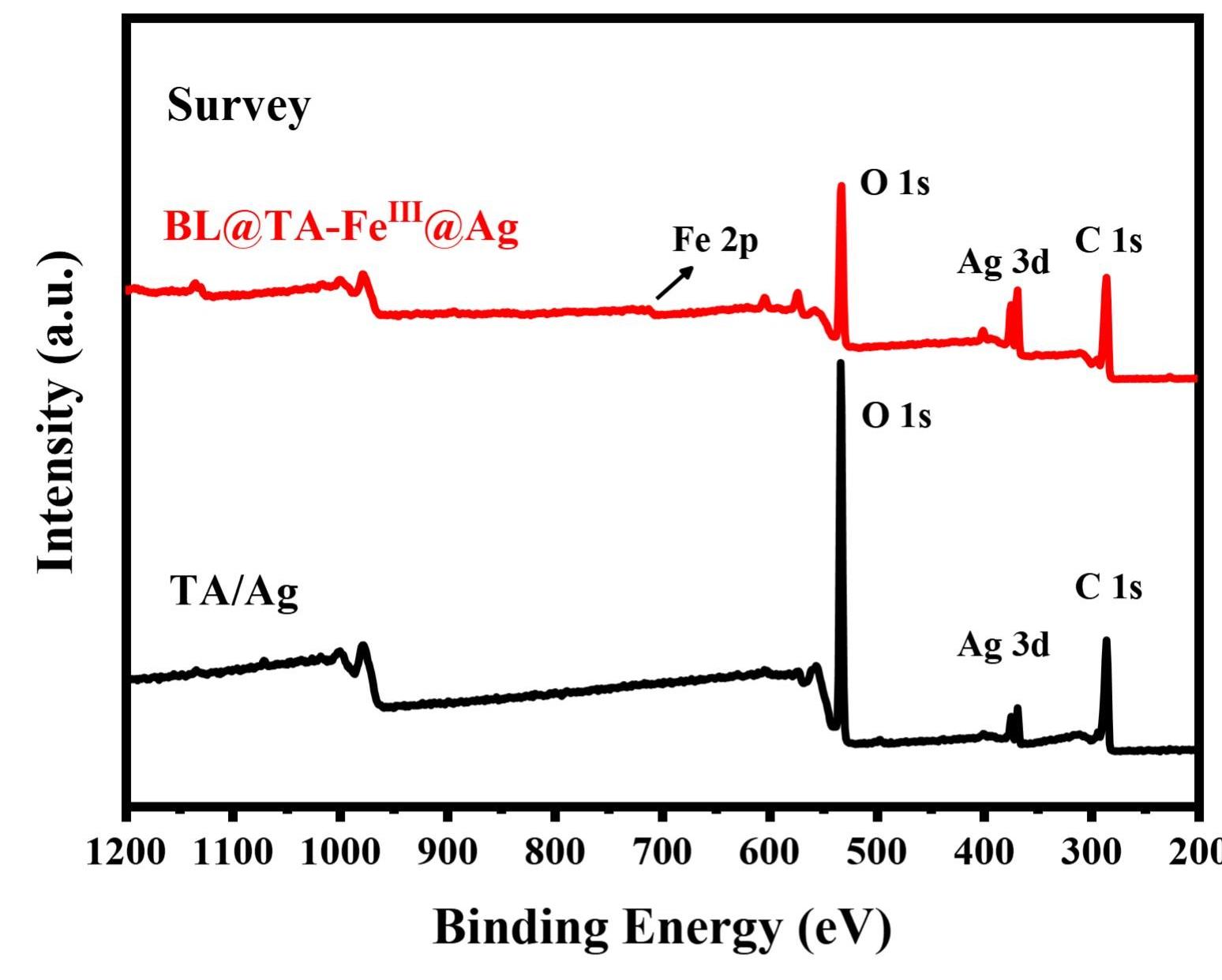


**Fig. S2** XPS profile analysis of BL@TA-Fe^III^@AgNPs and TA-Fe^III^@AgNPs.


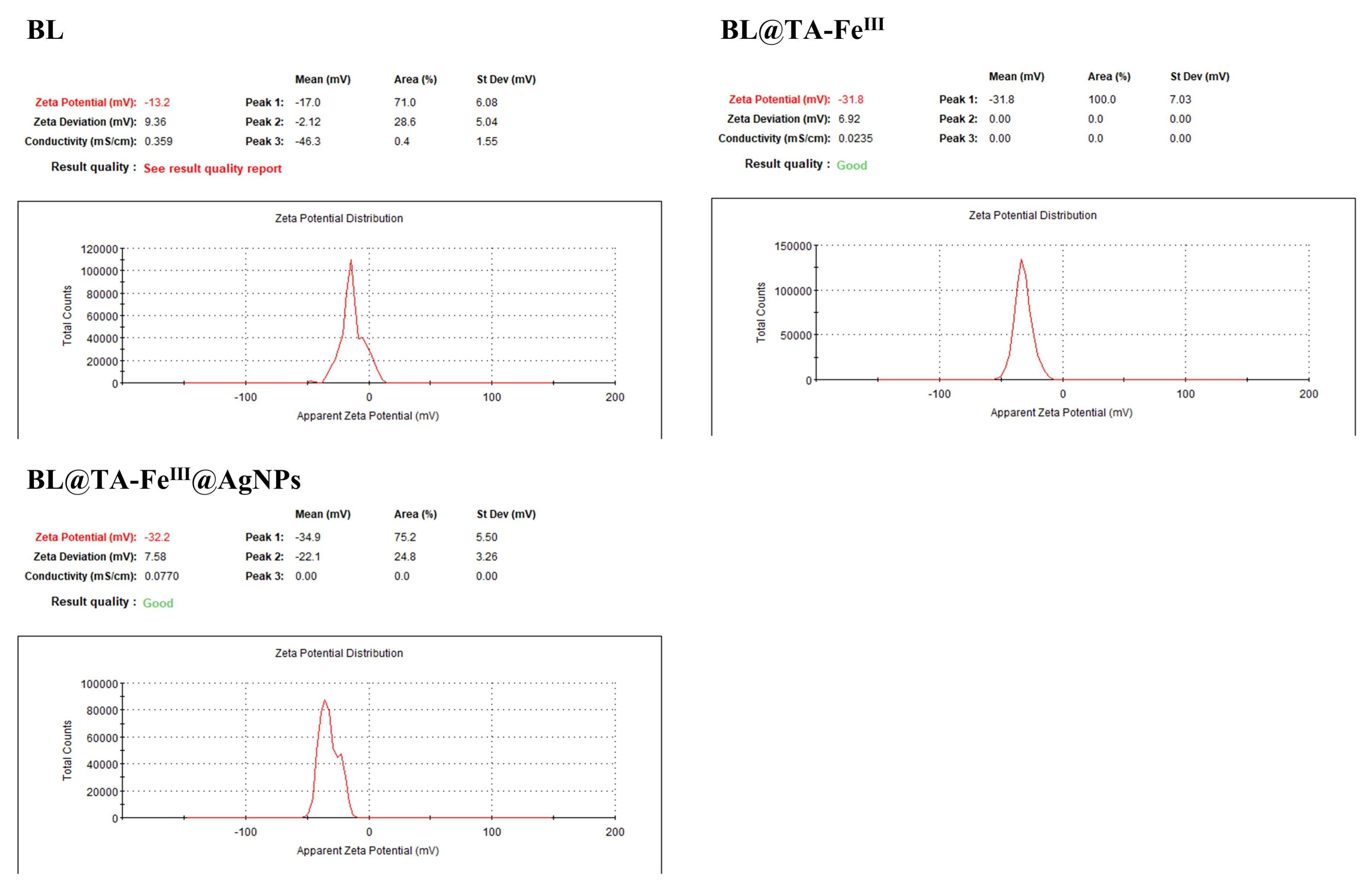


**Fig. S3** Zeta potential analysis of BL, BL@TA-Fe^III^, and BL@TA-Fe^III^@AgNPs suspensions.

**Fig. S4** **Full-wavelength UV-Vis absorption spectra of TA-Fe^III^ shells loaded or unloaded with AgNPs.**


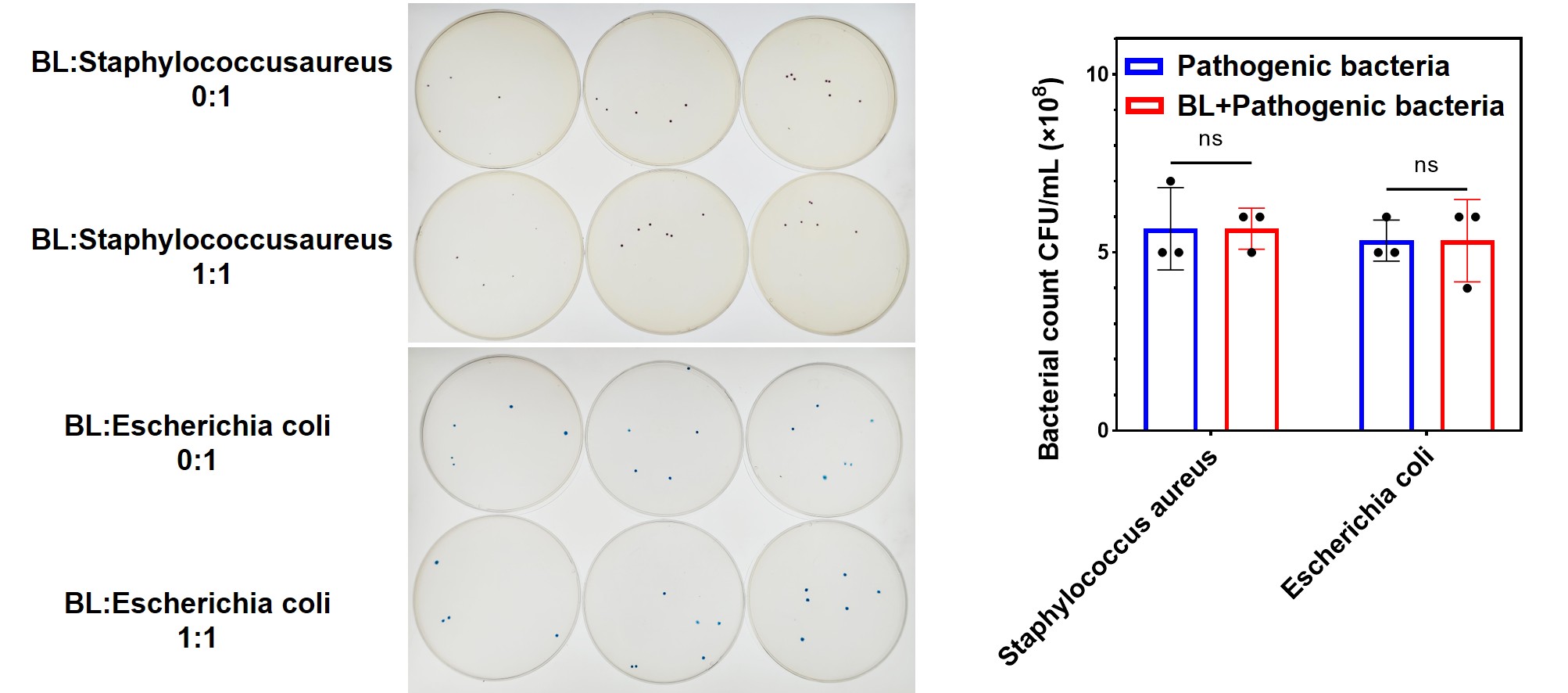


**Fig. S5** **BL direct effect on escherichia coli and staphylococcus aureus.**


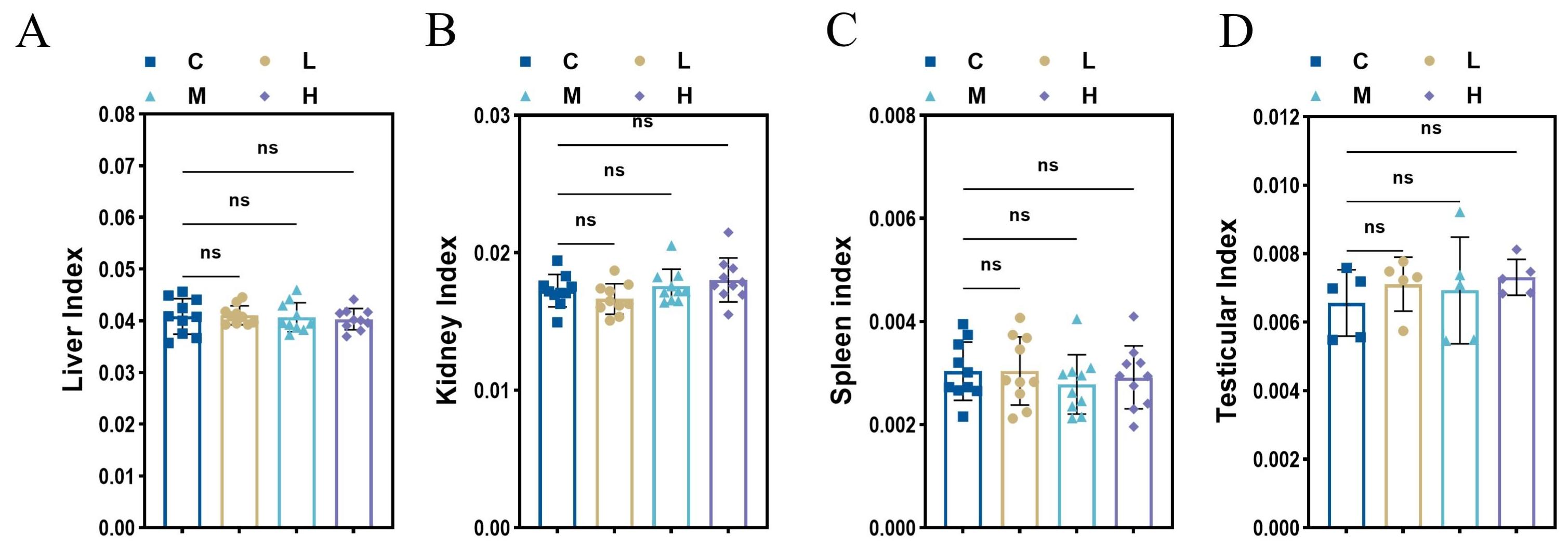


**Fig. S6** Effects of different doses of BL@TA-Fe^III^@AgNPs on organ indices of mice. (A) Liver index. (B) Kidney index. (C) Spleen index. (D) Testis index. Significant variations had been denoted with the aid of * (P < 0.05), ** (P < 0.01), or *** (P < 0.001).


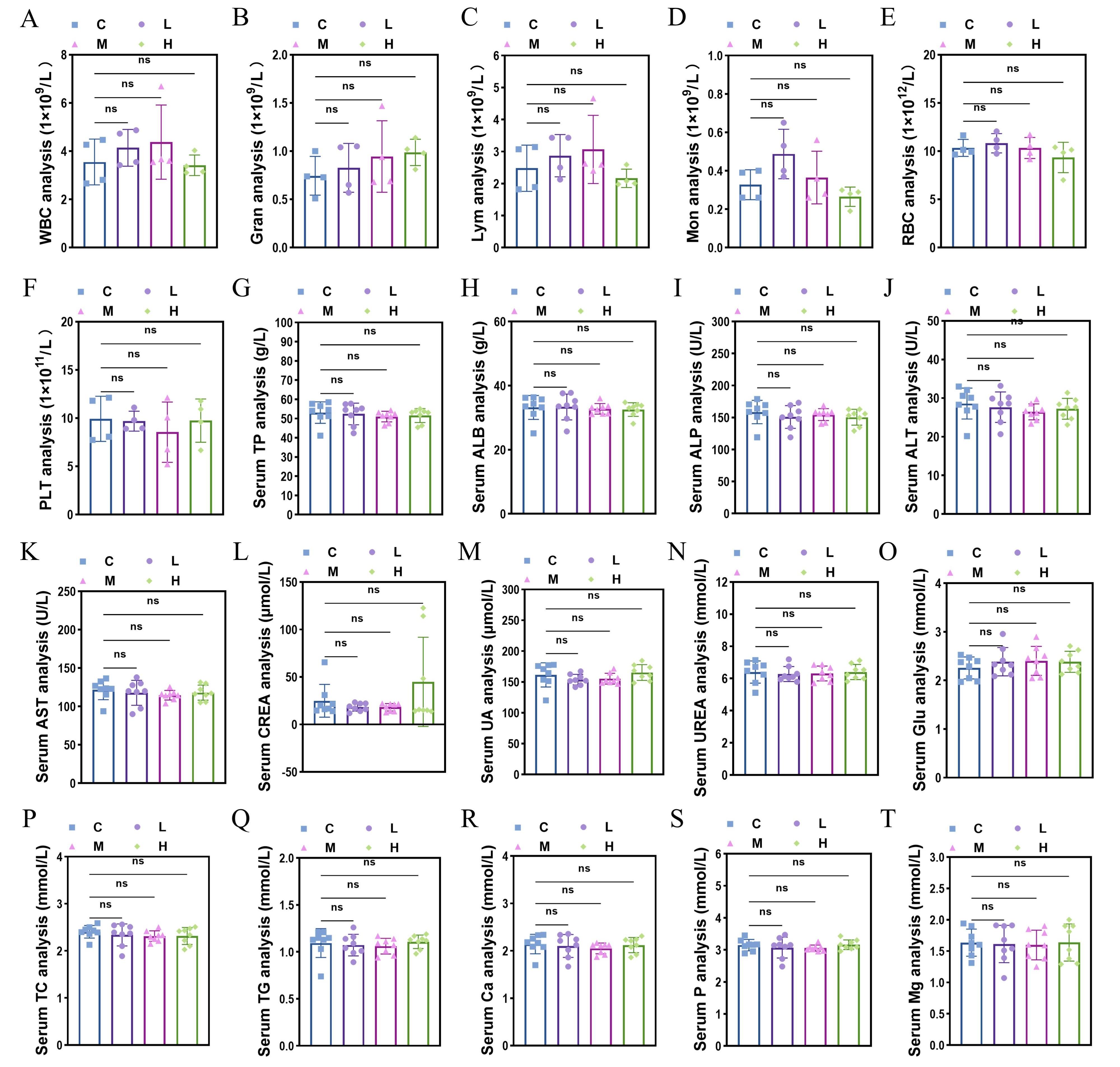


**Fig. S7** Effects of different doses of BL@TA-Fe^III^@AgNPs on blood and serum biochemical parameters of mice. Blood composition analysis includes WBC, Gran, Lym, Mon, RBC, PLT (A-F). Serum biochemical indicators related to liver function include TP, ALB, ALP, ALT, AST (G-K). Indicators related to renal function include UREA, CREA, UA (L-N). Indicators of nutrient level include serum Glu, TC, TG (O-Q). Indicators related to electrolyte assessment include serum Ca, serum P, serum Mg (R-T). Significant variations had been denoted with the aid of * (P < 0.05), ** (P < 0.01), or *** (P < 0.001).

The cellular composition test in whole blood includes white blood cell count (WBC), granulocyte count (Gra), lymphocyte count (Lym), monocyte count (Mon), red blood cell count (RBC) and platelet count (PLT). Serum biochemical tests include the following: liver function-related indicators, such as serum total protein (TP), albumin (ALB), alkaline phosphatase (ALP), alanine aminotransferase (ALT), and aspartate aminotransferase (AST); renal function-related indicators, including urea (UREA), creatinine (CREA), and uric acid (UA); serum glucose (Glu), total cholesterol (TC), and triglycerides (TG); and also indicators to assess electrolyte levels, such as serum calcium (Ca), serum phosphorus (P), and serum triglycerides (TG). ), total cholesterol (TC), and triglycerides (TG); in addition, indicators to assess electrolyte levels such as serum calcium (Ca), serum phosphorus (P), and serum magnesium (Mg). BL@TA-Fe^III^@AgNPs did not significantly affect the blood and serum biochemical indices of mice at different doses, suggesting that the composite has a good biosafety in the evaluated dose range.


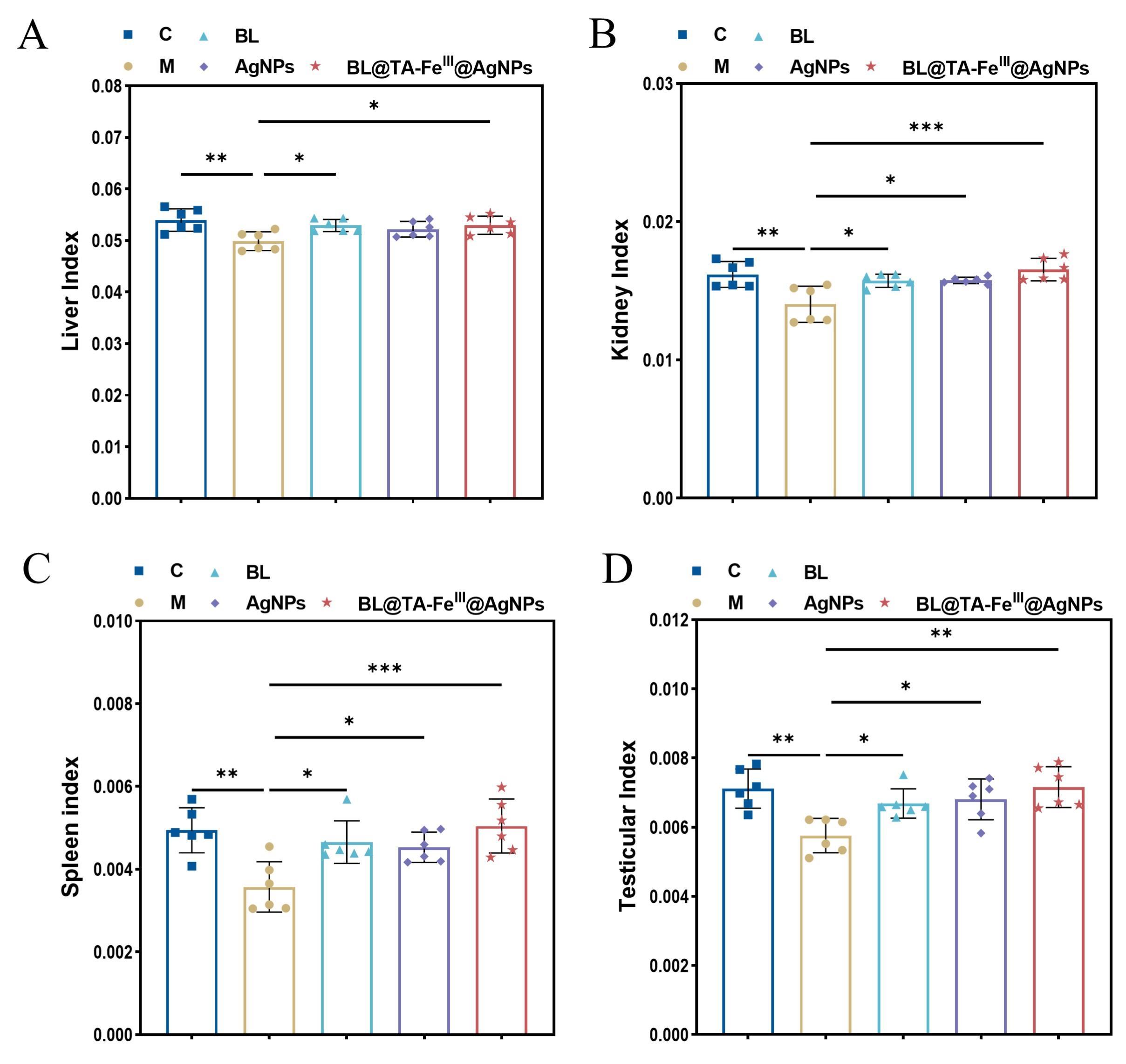


**Fig. S8** BL@TA-Fe^III^@AgNPs restore the decline in organ indices induced by LPS in mice (n=6). (A) Liver index. (B) Kidney index. (C) Spleen index. (D) Testis index. Significant variations had been denoted with the aid of * (P < 0.05), ** (P < 0.01), or *** (P < 0.001).


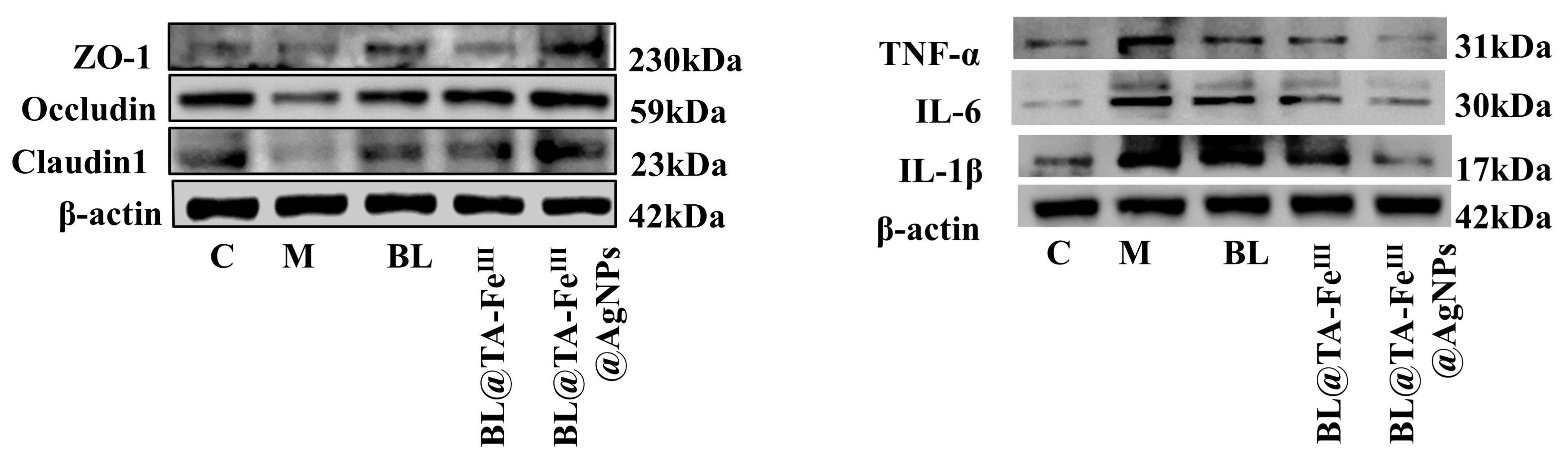


**Fig. S9** BL@TA-Fe^III^@AgNP improve the decrease in intestinal barrier protein expression and the increase in pro-inflammatory factor expression induced by LPS.


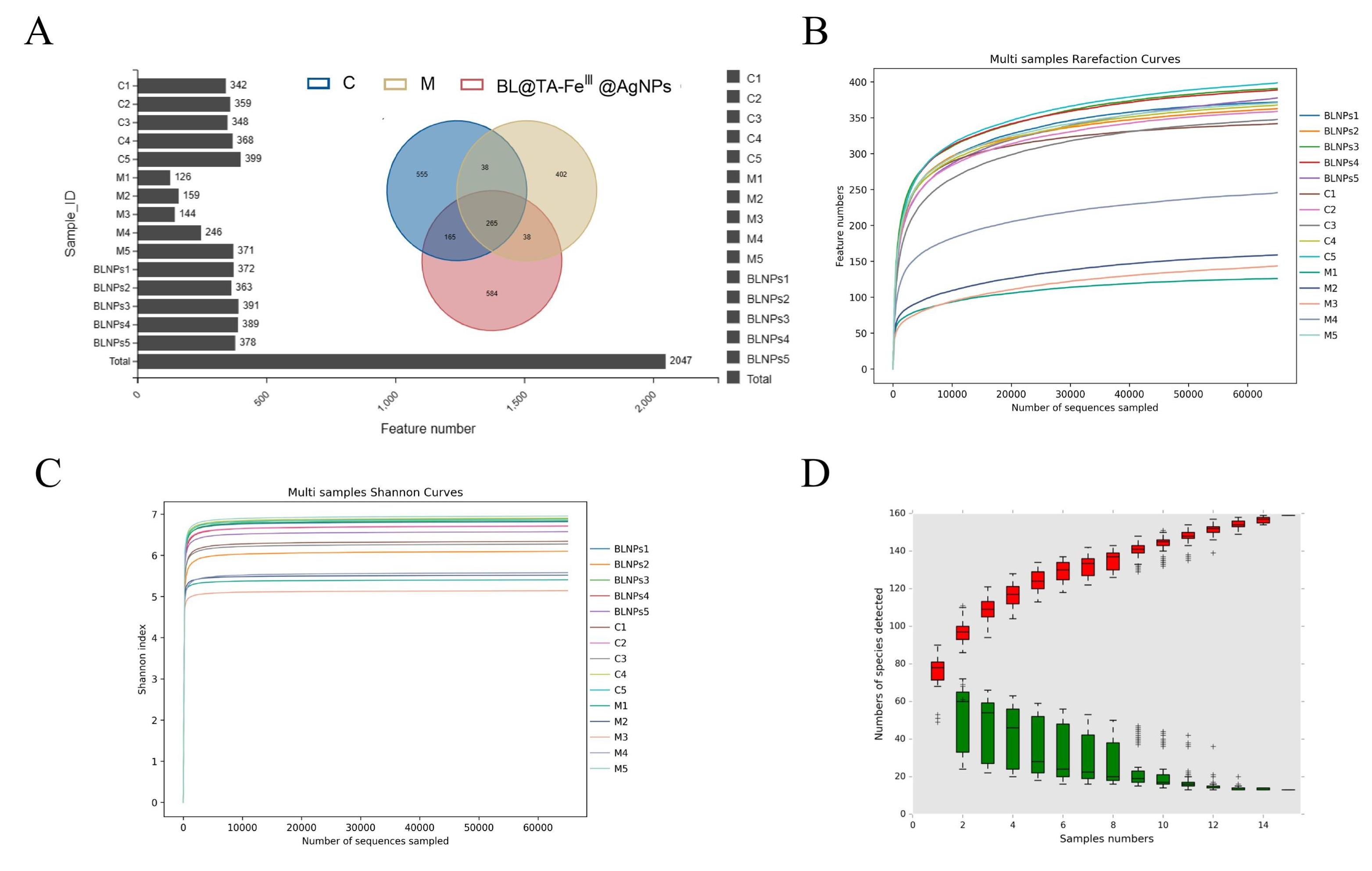


**Fig. S10** Assessment of the availability of intestinal microbiome sequencing data (n=5). (A) Number of OTU features in the samples and Venn diagram of intergroup features. (B) Rarefaction curve. (C) Shannon index curve. (D) Species accumulation curve.


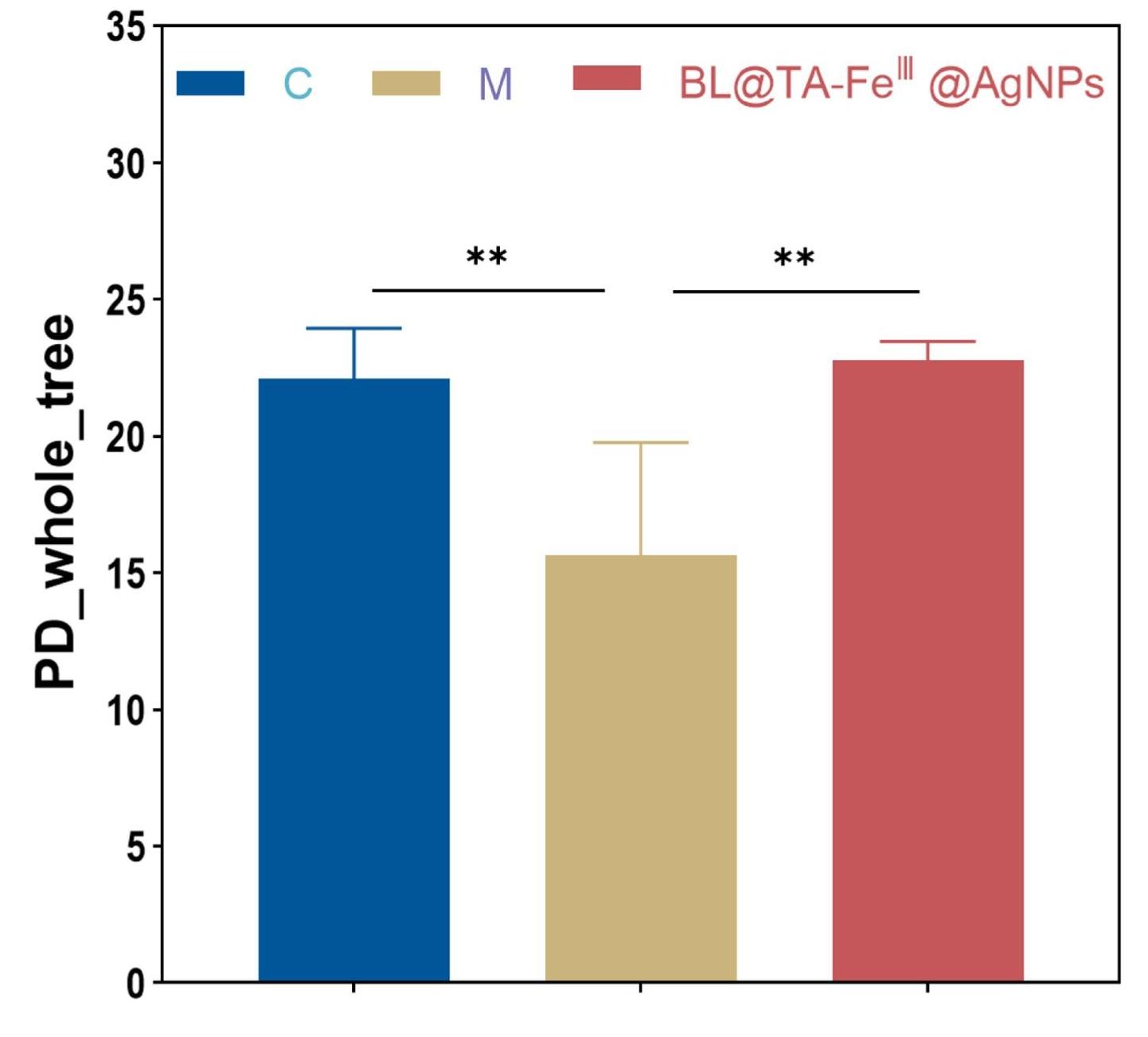


**Fig. S11** Diversity index analysis based on phylogenetic tree calculations. Significant variations had been denoted with the aid of * (P < 0.05), ** (P < 0.01), or *** (P < 0.001).
